# Persistence of viral full-length genomic RNA and replication-associated RNA transcripts and proteins in sheep tissues following acute Crimean-Congo hemorrhagic fever virus infection

**DOI:** 10.1371/journal.pntd.0014842

**Published:** 2026-10-30

**Authors:** Hongzhao Li, Estella Moffat, Katherine Handel, Michelle Nebroski, Daniel Sullivan, Andre Dufresne, Carissa Embury-Hyatt, Oliver Lung, Bradley S. Pickering

**Affiliations:** 1 National Centre for Foreign Animal Disease, Canadian Food Inspection Agency, Winnipeg, Manitoba, Canada; 2 Department of Medical Microbiology and Infectious Diseases, Faculty of Health Sciences, College of Medicine, University of Manitoba, Winnipeg, Manitoba, Canada; Hokkaido University: Hokkaido Daigaku, JAPAN

## Abstract

Crimean-Congo hemorrhagic fever virus (CCHFV) is a tick-borne RNA virus responsible for a severe hemorrhagic disease in humans. In contrast, the virus appears to cause only a brief viremia without overt clinical signs in animals such as livestock, following which the infection is traditionally assumed to end with complete viral clearance. Here, we challenge this prevailing view by reporting the recovery of full-length viral genomic RNA sequences from tissues of CCHFV-infected sheep long after the resolution of acute viremia. While viral RNA persistence in these tissues was largely characterized by a lack of extensive viral replication, we detected the sporadic presence of replication-associated viral RNA transcripts and viral proteins. These findings suggest that the biology of CCHFV in animals extends well beyond an acute infection. The possibility that the virus can transition into a dormant state, with potential for reactivation to resume gene expression or replication, a phenomenon documented in other RNA viruses, warrants further investigation.

## Author summary

Crimean–Congo hemorrhagic fever virus (CCHFV) is a tick‑borne virus that can cause a dangerous bleeding disease in humans. Livestock can also become infected and contribute to viral spread while showing no signs of illness. Traditionally, infected animals are believed to experience only a short period (a few days) of viral presence in their blood, after which the virus is thought to be completely cleared, leaving no viral footprint in the body. Our study challenges this long‑held assumption. In experimentally infected sheep, we found that intact CCHFV genetic material remains in lymphoid tissues weeks after the brief infection in the bloodstream has ended. This occurs without extensive production of infectious virus, while rare traces of viral RNA or protein associated with replication activities can still be found. These features suggest that the virus enters a predominantly silent, dormant‑like state possibly with potential for reactivation, rather than being fully eliminated. Our findings reveal that CCHFV can establish an extended, hidden phase of infection in livestock, which may influence how the virus is maintained and transmitted.

## Introduction

CCHFV is a risk group 4 virus and one of the top priority pathogens listed by the World Health Organization in urgent need for research and development [1–4]. Infection in humans can lead to a range of disease outcomes, including a severe, often fatal, acute hemorrhagic fever disease, characterized by vascular dysfunction, hemorrhagic manifestations, multi-organ failure, shock and death [1,5–7]. Case fatality rates are high, ranging typically from 5% to 30% and reportedly up to 80% in some outbreaks [1,8–10]. However, no internationally licensed vaccines or therapeutics are currently available against CCHFV. Maintained and transmitted primarily by ticks from the genus *Hyalomma*, the virus is endemic in countries across Asia, Europe and Africa, with emerging signs of geographic expansion and rising global incidence of infection [11,12].

CCHFV belongs to the genus *Orthonairovirus*, family *Nairoviridae*, order *Hareavirales,* Class *Bunyaviricetes* [13]. The virion contains a tri-segmented, single-stranded, negative-sense RNA genome (genomic vRNA) [14,15]. The small (S), medium (M) and large (L) genomic segments encode the nucleoprotein (N protein), glycoproteins and RNA-dependent RNA polymerase (RdRp), respectively. Genomic vRNA, N protein and RdRp together form the genomic ribonucleoprotein complexes (RNPs) inside an envelope with embedded glycoproteins. During viral replication, genomic vRNA is transcribed by RdRp to generate messenger RNA (mRNA) and genomic complementary RNA (genomic cRNA or antigenomic RNA), both of which are positive-sense. mRNA is translated into viral proteins, whereas genomic cRNA, assembling into antigenomic RNPs with N protein and RdRp, subsequently serves as a template for genomic vRNA replication [15]. In each genomic vRNA or cRNA segment, intra-strand base-pairing between the complementary terminal non-coding regions contributes to a panhandle-like secondary structure and a circular genomic appearance [15].

CCHFV circulates in a tick-vertebrate-tick cycle [1,16]. Ticks are the primary vector and long-term reservoir of the virus. A variety of wild and domestic animals can be productively infected and develop a viremia typically lasting a few days, without showing obvious signs of disease. While earlier experimental infection studies together with serological surveys have established a prominent role of animals as intermediate hosts for CCHFV maintenance and transmission, the investigations have been limited to the assessment of viremia and antibodies. The biology of CCHFV infection in animals remains poorly understood in many aspects. Among major unknowns are viral dissemination and persistence potentially occurring in tissues of infected animals [16].

For many decades, the traditional view holds that the short period of CCHFV viremia is followed by full recovery, with the virus completely eliminated and no lasting presence in the host. However, results from our recent experimental infection study in sheep challenge this assumption. Partial sequences of the S segment remained detectable in various tissues several weeks after the acute phase of viremia had ended [12], which points to a more prolonged virus–host interaction than previously appreciated. We therefore hypothesized a post-acute persistence of intact CCHFV genomic RNA sequences in tissues, potentially retaining the capacity to support the expression of viral transcripts and proteins associated with viral replication. Supporting this possibility, a human case report documented the presence of CCHFV RNA in tissues from a spontaneous abortion nine weeks after maternal infection, thereby extending the recognized duration for viral RNA detection in humans [17]. Furthermore, consistent with findings in CCHFV, similar phenomena of viral RNA persistence after the clearance of acute infection have been reported in other non-retroviral RNA viruses, such as measles (MeV), Ebola (EBOV), Marburg, Lassa, severe acute respiratory syndrome coronavirus 2 (SARS-CoV-2), tick-borne encephalitis (TBEV), Japanese encephalitis (JEV), Zika (ZIKV), West Nile (WNV), vesicular stomatitis (VSV) and Semliki Forest (SFV) viruses [18–31]. Viral RNA persistence has profound clinical and public health implications, ranging from long-term sequelae and late recrudescence to renewed viral transmission. In human survivors of acute EBOV infection, viral reactivation from a dormant state of persistence has caused severe/fatal disease and sparked new outbreaks [32–38]. Moreover, the induction of viral reactivation has been documented in animal models of persistence for MeV, SFV, Sindbis virus (SINV), WNV, JEV and TBEV [18,19,24,30,31,39–41].

However, due to technical challenges, our earlier efforts during the experimental infection study described above were unable to conclusively determine whether full-length CCHFV genomic RNA and molecular markers of viral replication persisted in post-acute sheep tissues [12]. In the present study, we revisited archived tissue samples from that prior infection study using newly developed approaches and obtained evidence supporting the long-term persistence of viral whole genome RNA sequences, accompanied by the presence of replication-associated viral transcripts and proteins. These data strengthen the possibility that the course and impact of CCHFV infection in animals may last longer than previously recognized.

## Results

### Signs of potential CCHFV persistence in sheep tissues from a previous study

CCHFV persistence was investigated using tissues collected during our recent experimental infection study, in which four domestic sheep (male and approximately two months old) were infected with the CCHFV Kosovo Hoti strain via both subcutaneous and intravenous routes at a dose of 1.2 × 10^6^ TCID50 [12]. These sheep all developed a brief viremia, which fell within 2 – 6 days post-infection (DPI) and remained negative for several weeks throughout later time points of the study (Fig 1A). In this context, potential viral dissemination and long-term persistence were evaluated using tissues collected on 34 DPI (Fig 1A). In the original study, partial sequences of the S segment were detected, primarily in lymph nodes and the spleen [12]. These observations then prompted further investigation into the nature of CCHFV RNA in these tissues, including whether the viral RNA retains the complete genome segments with a full coding capacity. It proved technically challenging, however, to detect from these tissues full-length genome segments or large portions of them, especially for the M and L segments. Conventional RT-PCR methods consistently generated negative results. We reason that this might have at least in part contributed to the lack of data in the literature on CCHFV genomic RNA in tissues of animals after recovery from acute infections.

**Fig 1:**
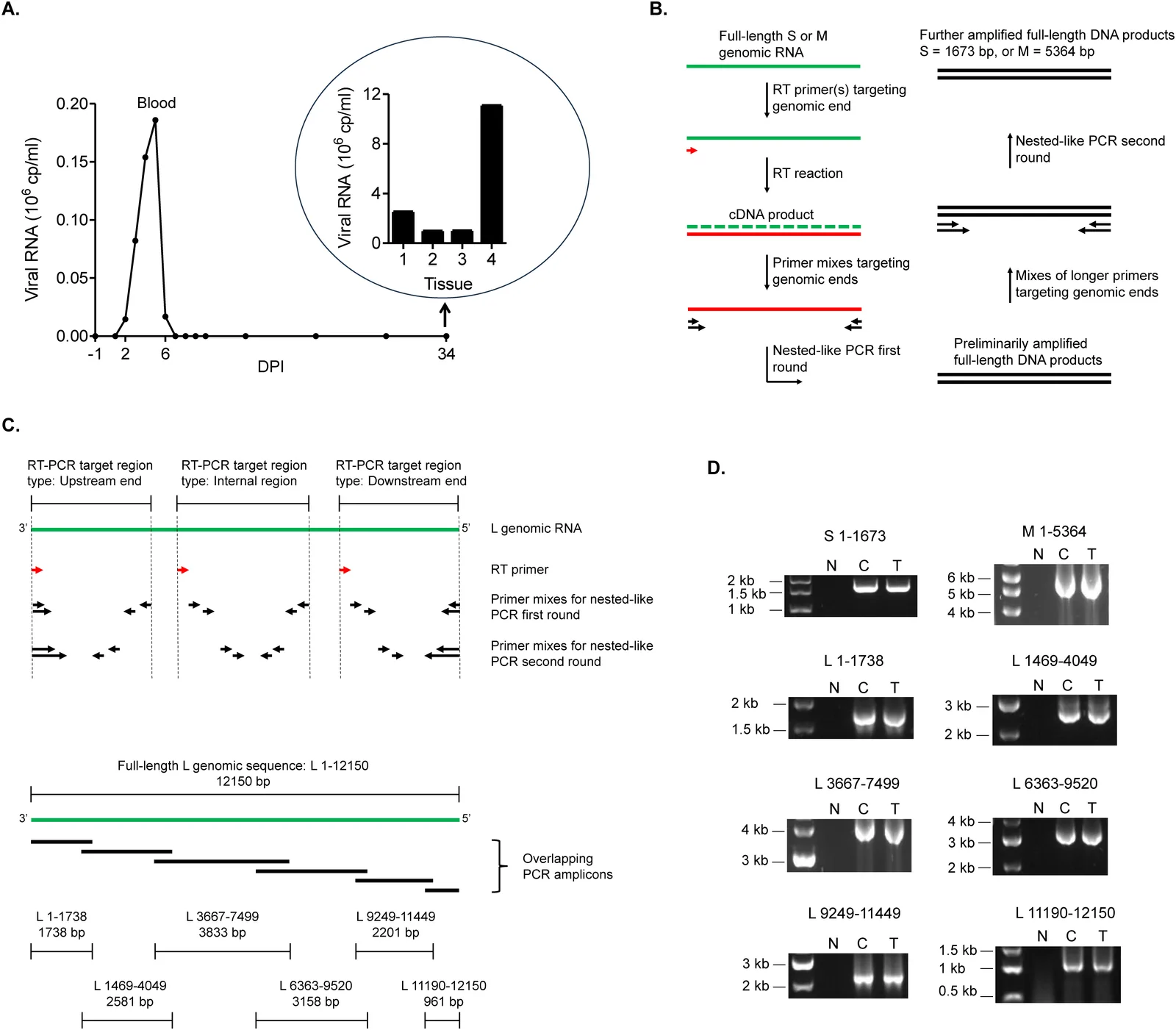
Post-acute CCHFV RNA persistence in sheep tissues. **A.** Illustration of potential viral persistence in tissues in the context of host recovery from acute viremia. Line graph shows mean viral RNA copy (cp) number/ml blood of a 57-nucleotide sequence of the S segment by RT-qPCR in four experimentally infected sheep (Sheep 1 through 4). Inset bar graph indicates the detection of this sequence in homogenates of several tissues on 34 DPI. Representative data are shown from four tissues. Tissue 1, Sheep 1 deep cervical lymph nodes (LN). Tissue 2, Sheep 2 inguinal LN; Tissue 3, Sheep 3 gastrohepatic LN. Tissue 4, Sheep 4 inguinal LN. **B.** Nested-like repeated PCR strategy to amplify full-length CCHFV S or M genomic RNA from sheep tissues. Repeated PCR rounds used primers targeting the ends of viral genomic segments. Primers of the second-round PCR are longer with a different 3’ sequence than those of the first round. A new “internal nesting” strategy was applied to each PCR round, in which a mixture of primers with various lengths and 3’ sequences were used to target each end of the genomic segment. bp, base pairs. **C.** Nested-like repeated PCR to amplify CCHFV L genomic RNA sequences from sheep tissues. The L segment due to its large size was amplified as overlapping amplicons. Repeated PCR rounds each with internal nesting were performed largely as in B with minor variations: **Top.** Diagram illustrates three types of amplicons based on their genomic location. Primers targeting extreme genomic ends were designed similarly to those in **B.** Primers targeting genomic internal sites did not share any sequence overlap, as in traditional nested PCR. **Bottom.** Genomic locations of the overlapping L amplicons. **D.** Full-length CCHFV genomic sequences were recovered from four different sheep tissues collected on 34 DPI, including Sheep 1 deep cervical LN, Sheep 2 inguinal LN, Sheep 3 gastrohepatic LN and Sheep 4 inguinal LN. Amplicon name above agarose gel of PCR products indicates target region on the genomic segment. N, negative control. C, cell culture of CCHFV. T, tissue, with PCR band of one tissue sample representing results from multiple sheep. PCR products were purified and sequenced to recover whole genome sequences.

### Technical advances led to the recovery of CCHFV whole genome RNA sequences persisting in tissues

In the present study, we used archived tissue samples from the prior experimental infection study described above to conduct new investigations into CCHFV persistence. We developed new, nested-like repeated PCR strategies to address challenges in the amplification of long templates with low copy numbers from a complex tissue background (Fig 1B and 1C). Among these was an “internal nesting” to overcome the outcompeting of long, intended amplicons by short, non-specific amplicons. We established numerous RT-PCR formulations, conditions and primer sets to effectively amplify different genomic RNA sequences (Methods and Table A in S1 File). As a result, the whole CCHFV genome was successfully amplified from lymph node tissues, including the full-length S and M segments each as a single amplicon and L segment as overlapping amplicons (Fig 1D), and sequenced by NGS methods (Table B in S1 File). It is important to note that viral RNA persistence in lymphoid tissues has been documented in other non-retroviral RNA viruses including MeV [24], TBEV [26,27], JEV [42], ZIKV [43], enterovirus [44], lymphocytic choriomeningitis virus [45], VSV [28,29] and SARS-CoV-2 [20–23].

We then performed multiple sequence alignment analysis comparing the tissue-derived viral sequences against both the Kosovo Hoti reference sequences from GenBank and the sequences of the original stock virus used to infect animals. No recurrent mutations were identified (Table B in S1 File), suggesting that CCHFV RNA persistence in tissues may be an inherent capability encoded in the innate viral sequences, independent of acquiring new functions through certain genomic mutations.

### Viral RNA persistence in tissues was not associated with the continuous, extensive presence of infectious virions

We next asked whether CCHFV RNA persistence in tissues involves active viral replication. Some RNA viruses, namely hepatitis C virus (HCV) and Borna disease virus (BDV), are known to persist by continuous viral replication while evading immune clearance [24]. In a variety of other RNA viruses, however, viral RNA can persist without the concurrent production of infectious virions, except during occasional, late reactivation events as reported in some of these viruses [24]. We conducted virus isolation, with our standard method of applying supernatants of tissue homogenates to SW-13 cell culture [12], as well as electron microscopy (Supplementary methods and Fig A in S1 File), but failed to detect infectious virus or whole virus morphology. While these results suggest a lack of infectious virions in the tissues of the current state, they do not definitively rule out the possibility that tissues harboring persistent CCHFV RNA may retain the capacity to support inducible viral reactivation. Addressing this possibility will likely require the application of more suitable and sensitive methodologies.

### Visualization of viral RNA persistence in tissues revealed the presence of replication products

To evaluate directly in tissues the presence and distribution patterns of potential CCHFV RNA and protein, ISH targeting negative-sense or positive-sense viral RNA (derived from the M segment) and IHC targeting viral protein (the N protein) were developed and performed in lymph node and spleen tissues (Table C in S1 File). Both negative-sense and positive-sense viral RNA populations were observed in multiple tested tissues (Table C in S1 File and Fig 2). Viral protein was detected in one of the lymph node tissues (Table C in S1 File and Fig 2). Without widespread presence, the viral RNA and protein demonstrated a sporadic localized distribution (Figs B-D in S1 File). The presence of viral positive-sense RNA and protein indicates the occurrence of recent viral gene expression or replication activities in tissues and suggests a retained capacity of the persistent viral genomic RNA in supporting such activities. Finally, we provide a hypothetical model in which we summarize the current findings and propose possible mechanisms supporting the establishment and maintenance of CCHFV RNA persistence in tissues (Fig E in S1 File). In this context, we highlight a need for future investigation into the potential of viral reactivation harbored in such persistence.

**Fig 2:**
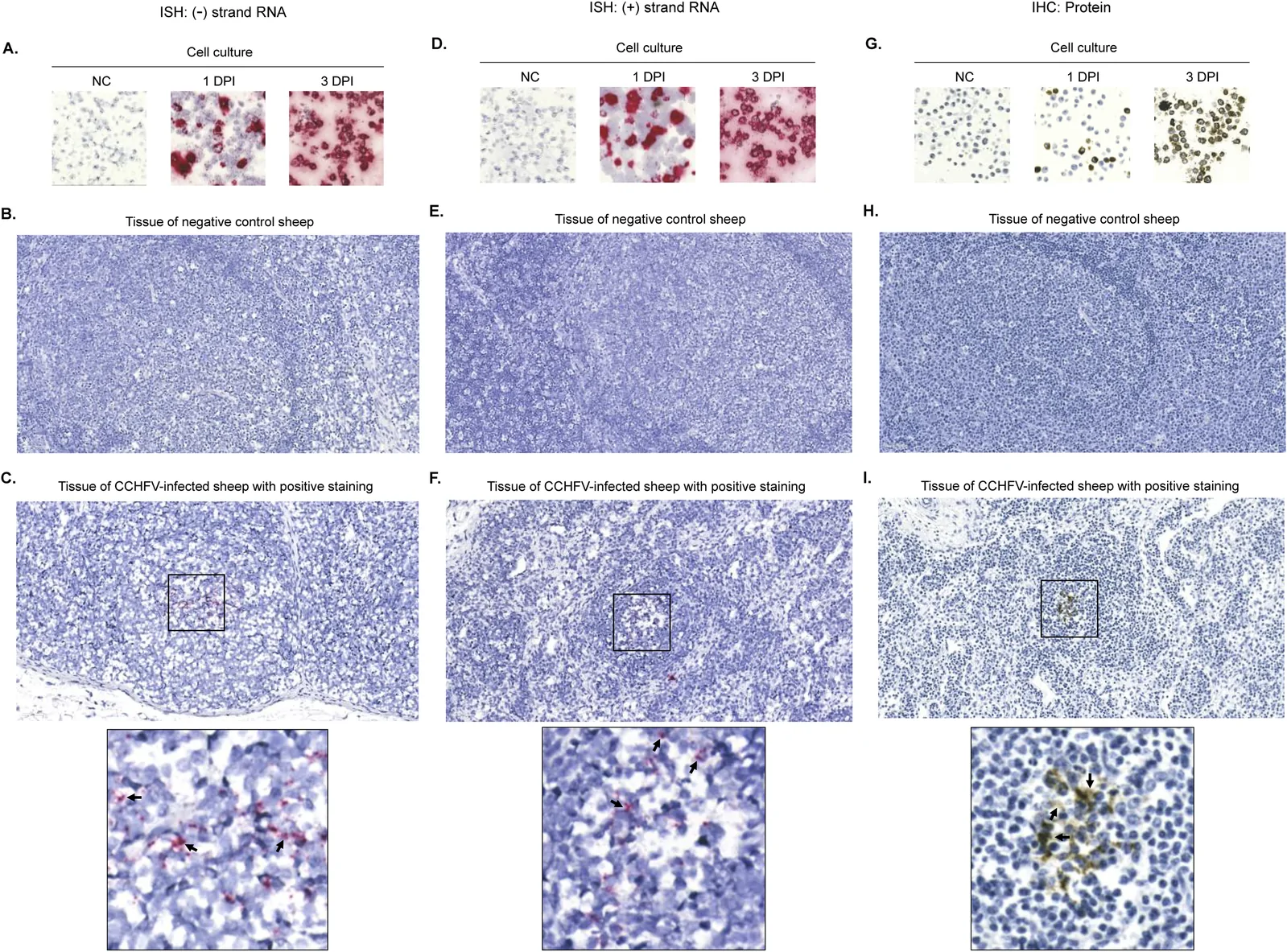
Visualization of CCHFV RNA and protein in sheep lymphoid tissues. Negative-sense (A-C) and positive-sense (D-F) viral RNAs (M segment) were detected by situ hybridization (ISH) and viral protein (N protein, G-I) by immunohistochemistry (IHC). **A, D and G**. Images of control staining using cell pellets from CCHFV cell cultures. NC, negative control cell culture without CCHFV. 1 DPI and 3 DPI, positive control cell cultures with CCHFV on 1 and 3 day(s) post-infection, respectively. **B, E and H.** Images of CCHFV-negative control staining using a lymph node tissue from a sheep infected with foot-and-mouth disease virus. **C, F and I.** Images representing positive staining in lymph node tissues from CCHFV-infected sheep (34 DPI). Enlarged illustration of area highlighted with square is displayed at the bottom. Arrows point to representative areas of positive staining. Negative-sense viral RNA was detected in lymph nodes from four sheep and the spleen from one sheep, positive-sense viral RNA was detected in lymph nodes from four sheep, and viral protein was detected in a lymph node from one sheep. A summary of these findings across all tissues examined has been provided in Table C in S1 File.

## Discussion

The detection of a CCHFV sequence from sheep tissues by RT-qPCR long after acute infection (Fig 1A) may lead to the assumption that this represents remnants of infection in the form of fragmented or degraded viral RNA. However, such nonfunctional remnants would be unlikely to persist for those several weeks, as RNA is highly susceptible to rapid degradation and clearance in the absence of cellular protective mechanisms. It has been observed in several RNA viruses that long-term dormant viral forms possess the potential to resume mRNA transcription, protein synthesis and infectious virus production, attesting to the persistence of full-length genomic RNA [18,19,24,30,31,38,39,46,47]. Consistent with this, we recovered CCHFV full-length S and M segments from sheep tissues. We were only able to obtain the whole sequence of the L segment using overlapping PCR amplicons. No method was available from the literature or our own custom development to amplify the L segment as a single amplicon, even with high-concentration purified template CCHFV RNA extracted from cell culture supernatants. Thus, we could not definitely confirm the full-length nature of the L segment sequences. Nevertheless, the recovery of full-length S and M segments strongly suggests the presence of a functional biological mechanism(s) required to maintain their long-term persistence in intact forms.

In addition to the possession of full-length genome segments, CCHFV RNA persistence in tissues exhibited other features: lack of culturable infectious virions, the presence of replication-associated viral RNA and protein, as well as a sporadic localized distribution of viral RNA and protein. These illustrate a largely dormant state without continuous and extensive production of infectious virions, but do not exclude the possibility of genomic replication (vRNA and cRNA) independent of a concurrent virion production or the possibility of sporadic viral replication. The lack of recoverable virus may also be attributed to an inadequate viral isolation method [24].

## Methods

### Animal work, ethics and biosafety approval statement

Archived sheep tissue samples from a recent experimental infection study [12] were used here to investigate CCHFV RNA persistence. All animal-related work was approved by the Animal Care Committee of the Canadian Science Centre for Human and Animal Health, under the animal user document number C-21–001. Experiments involving infectious CCHFV were all conducted in the containment level 4 laboratory at the National Centre for Foreign Animal Diseases, Canadian Food Inspection Agency, following the institutional standard operating procedures.

### Amplification of viral genomic RNA sequences from sheep tissue homogenates by reverse transcription (RT) and nested-like repeated polymerase chain reactions (nested-like repeated PCR)

Four lymph node (LN) tissues, with the highest positive signals for CCHFV RNA during a previous RT-qPCR screening [12], were analyzed for the presence of full-length viral genomic RNA sequences, including Sheep 1 deep cervical LN, Sheep 2 inguinal LN, Sheep 3 gastrohepatic LN and Sheep 4 inguinal LN. Sheep tissue homogenization, sample inactivation and RNA extraction were performed as previously described [12,48,49]. In-house RT-nested-like repeated PCR strategies were developed to improve amplification specificity and yield. These typically included two or three rounds of PCR reactions each involving a mix of multiple forward primers or/and a mix of multiple reverse primers. The RT reaction was conducted using Maxima H Minus First Strand cDNA Synthesis Kit (ThermoFisher Scientific, K1652) or ProtoScript II First Strand cDNA Synthesis Kit (New England Biolabs, E6560L). Repeated PCR rounds were performed using the LongAmp Taq PCR Kit (New England Biolabs, E5200S). In some cases, the RT and first round PCR were run as a one-step RT-PCR using SuperScript III One-Step RT-PCR System with Platinum Taq DNA polymerase (ThermoFisher Scientific, 12574018). Common RT-nested-like repeated PCR procedures are described below while amplicon-specific variations in reaction formulations, conditions and primer sequences are detailed in Table A in S1 File.

For RT reaction using the Maxima H Minus First Strand cDNA Synthesis Kit (ThermoFisher Scientific, K1652), a denaturation/annealing reaction (15 µl) containing 8 µl water, 1 µl 10 µM or 100 µM RT primer, 5 µl RNA extraction and 1 µl 10 mM dNTP mix was performed with the thermal program: Denaturation at 65 °C for 5 minutes and annealing at 4°C for 5 minutes. After addition of 4 µl 5 × RT Buffer and 1 µl Maxima H Minus Enzyme Mix, the final RT reaction (20 µl) was run with the thermal program: RT at 50 °C or 65 °C for 30 minutes, RT enzyme inactivation at 85 °C for 5 minutes and incubation at 4 °C for 5 minutes. For RT reaction using the ProtoScript II First Strand cDNA Synthesis Kit (New England Biolabs, E6560L), a denaturation/annealing reaction (8 µl) containing 2 µl water, 1 µl 10 µM RT primer and 5 µl RNA extraction was performed with the same thermal program as above with the Maxima H Minus RT. After addition of 10 µl 2 × ProtoScript II Reaction Mix and 2 µl 10 × ProtoScript II Enzyme Mix, the final RT reaction (20 µl) was run with the thermal program: RT at 42 °C for 60 minutes, RT enzyme inactivation at 85 °C for 5 minutes and incubation at 4 °C for 5 minutes.

For PCR rounds using the LongAmp Taq PCR Kit (New England Biolabs, E5200S), the first round PCR reaction (20 µl) consisted of 8 µl water, 4 µl 5 × LongAmp Taq Reaction Buffer, 0.6 µl 10 mM dNTP mix, 0.8 µl 10 µM (total primer concentration) forward primer or primer mix, 0.8 µl 10 µM (total primer concentration) reverse primer or primer mix, 0.8 µl LongAmp Taq and 5 µl template (RT products). The second or third round PCR reaction had the same formulation except that the volumes of water and template (previous round PCR products) were 12 µl and 1 µl, respectively. The thermal program for LongAmp Taq PCR was: pre-denaturation at 94 °C for 30 seconds, 40 amplification cycles (denaturation at 94 °C for 10 seconds, annealing at 65 °C for 1 minute and extension at 65 °C for a customized time depending on the amplicon size, largely 50 seconds/kb), final extension at 65 °C for 10 minutes and cooling down at 4 °C for 5 minutes. The final PCR products were visualized on a 0.8% agarose gel with the molecular size marker 1 kb DNA Ladder (New England BioLabs, N3232S).

For one-step RT-PCR (combining RT and the first round of nested PCRs) using the SuperScript III One-Step RT-PCR System with Platinum Taq DNA polymerase (ThermoFisher Scientific, 12574018), the reaction (50 µl) consisted of 16 µl water, 25 µl 2 × Reaction Mix, 2 µl SuperScript III RT/Platinum Taq Mix, 1 µl 10 mM forward primer mix, 1 µl 10 mM reverse primer mix and 5 µl RNA template. The thermal program was: RT at 52.5 °C for 30 minutes, pre-denaturation at 94 °C for 2 minutes, 40 amplification cycles (denaturation at 94 °C for 15 seconds, annealing at 50.5 °C for 30 seconds and extension at 68 °C for 1 minute 40 seconds), final extension at 68 °C for 5 minutes and cooling down at 4 °C for 5 minutes. 1 µl of the resulting RT-PCR products was used as the template in a subsequent 20 µl LongAmp Taq PCR reaction.

### Next Generation Sequencing (NGS) of CCHFV RNAs from sheep tissues

PCR amplicons were gel-purified using the MinElute Gel Extraction Kit (Qiagen, 28606), eluted with 10 – 20 µl water. The purified amplicons were either directly used or cloned into plasmids for sequencing using the TOPO TA Cloning Kit for Sequencing, with One Shot TOP10 Chemically Competent E. coli (ThermoFisher Scientific, K457540). Amplicons were sequenced using the Native Barcoding Kit 96 (Oxford Nanopore Technologies, ONT, SQK-NBD114.96) following the manufacturer’s protocol with recommended third-party reagents. The concentration of the amplicons was determined using a Qubit Fluorometer and a Qubit 1X dsDNA HS Assay Kit (ThermoFisher Scientific, Q33231). 200 fmol of DNA was used for NGS following the manufacturer’s protocol (ONT). Final 50 fmol pooled libraries were loaded onto R10.4.1 chemistry SpotON Flow Cells (FLO-MIN114) and sequenced on an ONT GridION device (ONT). Plasmids were sequenced using ONT or Illumina technology. For ONT, 5 ng of plasmid DNA was sequenced using the Rapid PCR Barcoding Kit (ONT, SQK-RPB114.24) following the manufacturer’s protocol, with 28 cycles of PCR and LongAmp Taq 2 × Master Mix (New England Biolabs, M0287). Concentration of the plasmids was determined using the Qubit method as above. 50 fmol of the final pooled library was sequenced as above. For Illumina sequencing, the concentration of the plasmids was determined as above. The plasmids were diluted to the appropriate concentration and libraries were prepared using the Nextera XT DNA Library Prep Kit (Illumina, FC-131–1096) and Nextera XT Index Kit v2 (Illumina, FC-131–2004), following the Illumina protocol. Libraries were cleaned up using AMpure XP Beads (Beckman Coulter) and were run on the Agilent TapeStation 4200 to determine the average fragment size. The library pool concentration was determined by the Qubit Fluorometer and the final 9 pM library was loaded onto a MiSeq v2, 300-cycle flow cell (Illumina, MS-102–2002) and sequenced on an Illumina MiSeq System.

### NGS sequencing data analysis

For Illumina sequencing runs, initial analysis was performed by running all samples through the CFIA-NCFAD/nf-villumina (https://github.com/CFIA-NCFAD/nf-villumina, v2.0.1) Nextflow (v24.04.2) [50] workflow for quality control. First, nf-villumina removed Illumina PhiX Sequencing Control V3 reads using BBDuk (v39.06) [51], followed by adapter removal and quality filtering with fastp (v0.23.4) [52]. QC filtered reads were mapped to the CCHFV Kosovo Hoti reference sequence in Geneious Prime (v2025.0.2) (https://www.geneious.com) using the Minimap2 (v.2.24) [53] assembler to generate a 75% majority consensus sequence with a minimum 10 × coverage threshold. A nucleotide position was assigned a non-degenerate base when a single nucleotide accounted for ≥75% of the sequencing reads at that position. If no nucleotide reached this 75% threshold, i.e., two nucleotides both fell within a frequency range of 25% - 75% (exclusive), the position was designated using a degenerate nucleotide code to reflect sequence variation. The final consensus sequences were aligned to the reference sequence with MAFFT (v7.490) [54] in Geneious Prime to determine pairwise identity.

For ONT sequencing runs, initial analysis was performed by re-basecalling the data with Dorado (https://github.com/nanoporetech/dorado, v0.9) and then performing quality trimming with Porechop_ABI (v0.5.0) [55]. In cases where large amounts of reads were produced, trimmed reads were subsampled down to 30,000 reads using seqtk (https://github.com/lh3/seqtk, v1.3-r106). Trimmed and subsampled reads were mapped against the designated CCHFV segment reference sequence in Geneious Prime (v2025.0.2) based on the target amplicon regions using Minimap2 (v2.24) [53]. Amplicon regions were determined from the final target amplicon lengths from repeated PCR rounds and 75% majority consensus sequences with a minimum 30 × coverage threshold were extracted from these regions. Degenerate nucleotides were determined using the criteria described above. Final consensus sequences were aligned back to the reference sequence in Geneious Prime using MAFFT (v7.490) [54] to determine pairwise identity.

### Immunohistochemistry (IHC) and in situ hybridization (ISH)

Tissue blocks were sectioned at 5 µm and stained with haematoxylin and eosin (HE) for histopathologic examination. For IHC, paraffin tissue sections were quenched for 10 minutes in aqueous 3% hydrogen peroxide. Epitopes were then retrieved using proteinase K for 10 minutes then rinsed. The primary antibody applied to the sections was a rabbit polyclonal Anti-Nucleoprotein Crimean-Congo hemorrhagic fever virus antibody (Abcam, ab190657) and was used at a 1:2,000 dilution overnight at 4 °C. Staining was then visualized using the Dako EnVision + /HRP system (anti-rabbit) (Agilent, K400311-2) and Liquid DAB+ (Agilent, K346811-2). The sections were then counter stained with Gill’s haematoxylin. For ISH, 5 µm paraffin-embedded formalin-fixed tissue sections were cut, air dried and melted onto charged slides in a 60 °C oven. The slides were then cleared and hydrated in xylene and 100% ethanol and air dried. The sections were quenched for 10 minutes in aqueous H_2_O_2_, boiled in target retrieval solution for 15 minutes, rinsed in 100% ethanol and air dried again. A final pre-treatment of protease plus enzyme for 30 minutes at 40 °C was applied. The RNAscope Probe, V-CCHFV-M-env-sense (Advanced Cell Diagnostics, 497341) or V-CCHFV-M-env (Advanced Cell Diagnostics, 479781) was applied and incubated at 40 °C for 2 hours. Hybridization amplification steps (AMP 1–6) were applied to the slides for the recommended times and temperatures as per the manual for the RNAscope 2.5 HD Reagent Kit - RED (Advanced Cell Diagnostics, 322350). The signal was visualized by the chromogen Fast Red. The sections were then counter stained with Gill’s 1 hematoxylin, dried and coverslipped. Microscopic images were acquired using the Aperio LVI real-time digital pathology system and Aperio ImageScope slide viewing software version 12.4.3.

## Supporting information

S1 File Supplementary results and discussion, methods, tables and figures. (DOCX)

S1 Raw Gel Raw gel images. (PDF)
