# Supplementary material for: Persistence of viral full-length genomic RNA and replication-associated RNA transcripts and proteins in sheep tissues following acute Crimean-Congo hemorrhagic fever virus infection
Source: PLoS Negl Trop Dis. 2026 Oct 30;20(10):e0014842. doi: 10.1371/journal.pntd.0014842 (PMC13734597; doi:10.1371/journal.pntd.0014842)
Supplement: Supplementary file 1 [file pntd.0014842.s002.pdf]

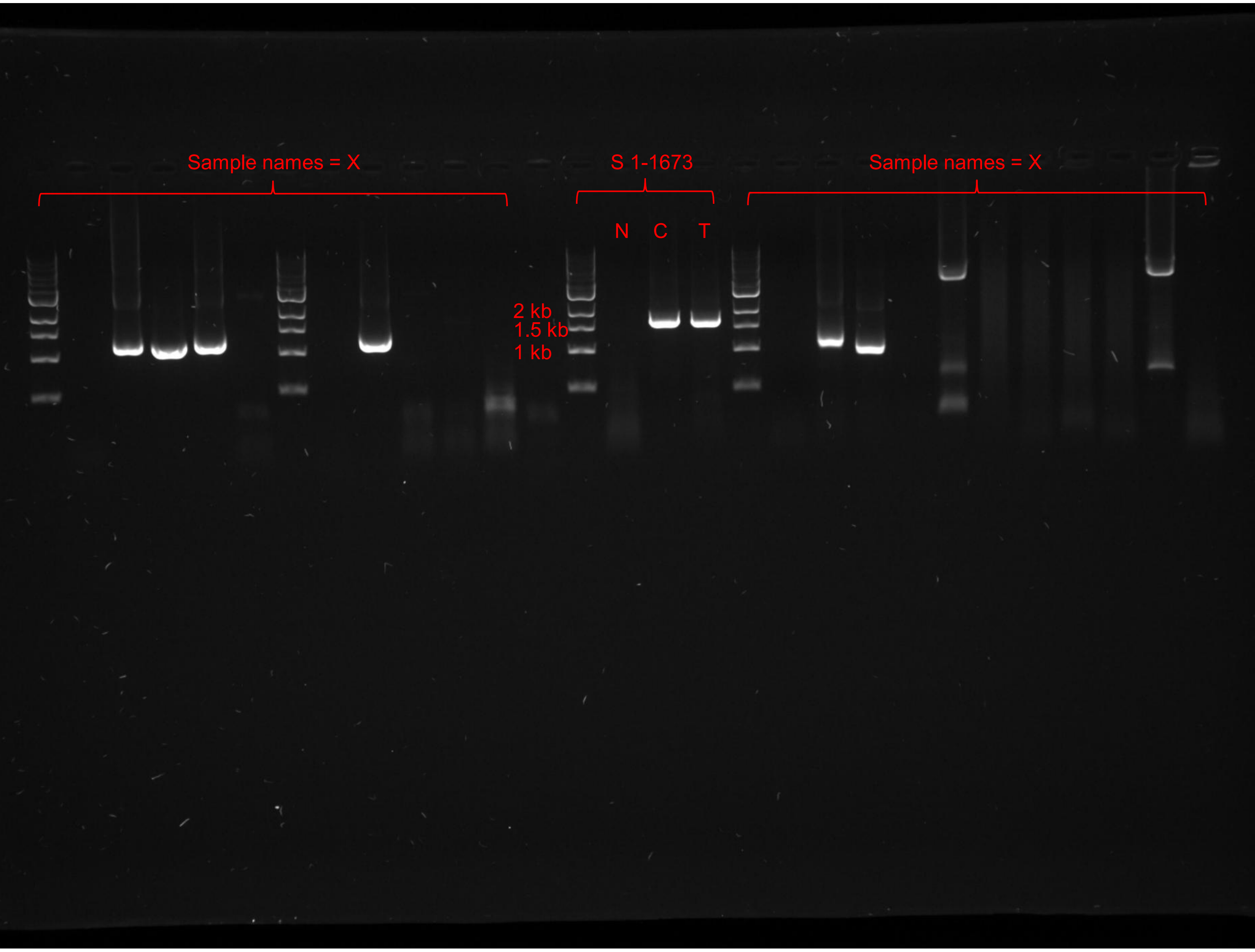

Raw agarose gel image for Fig 1D panel S1-1673. Lanes that were not included in the final figure are labelled with sample names "X". Agarose gel image was acquired on a BioRad ChemiDoc Imaging System using the Image Lab Touch Software v3.0.1.14.

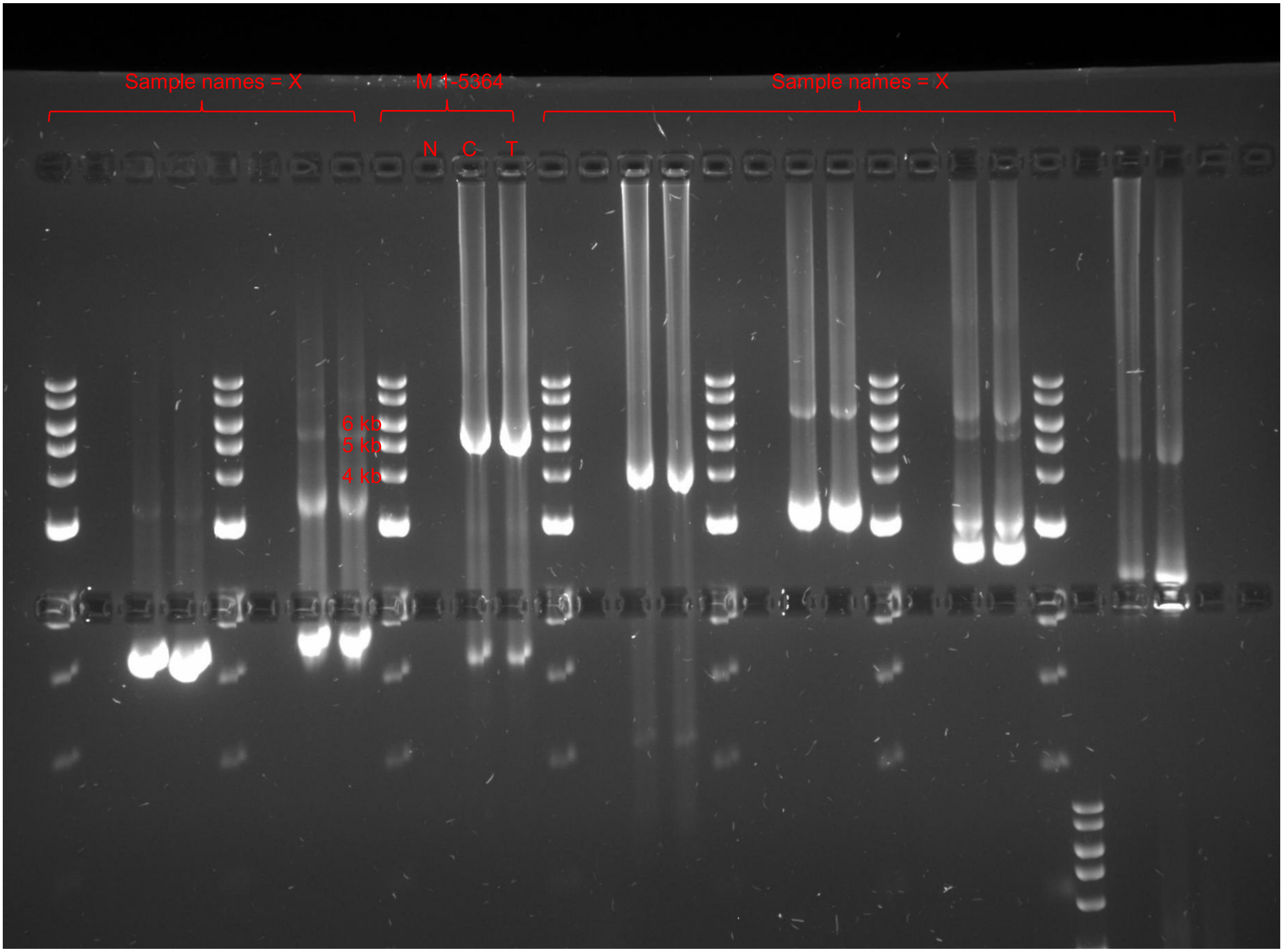

Raw agarose gel image for Fig 1D panel M 1-5364. Lanes that were not included in the final figure are labelled with sample names "X". Agarose gel image was acquired on a BioRad ChemiDoc Imaging System using the Image Lab Touch Software v3.0.1.14.

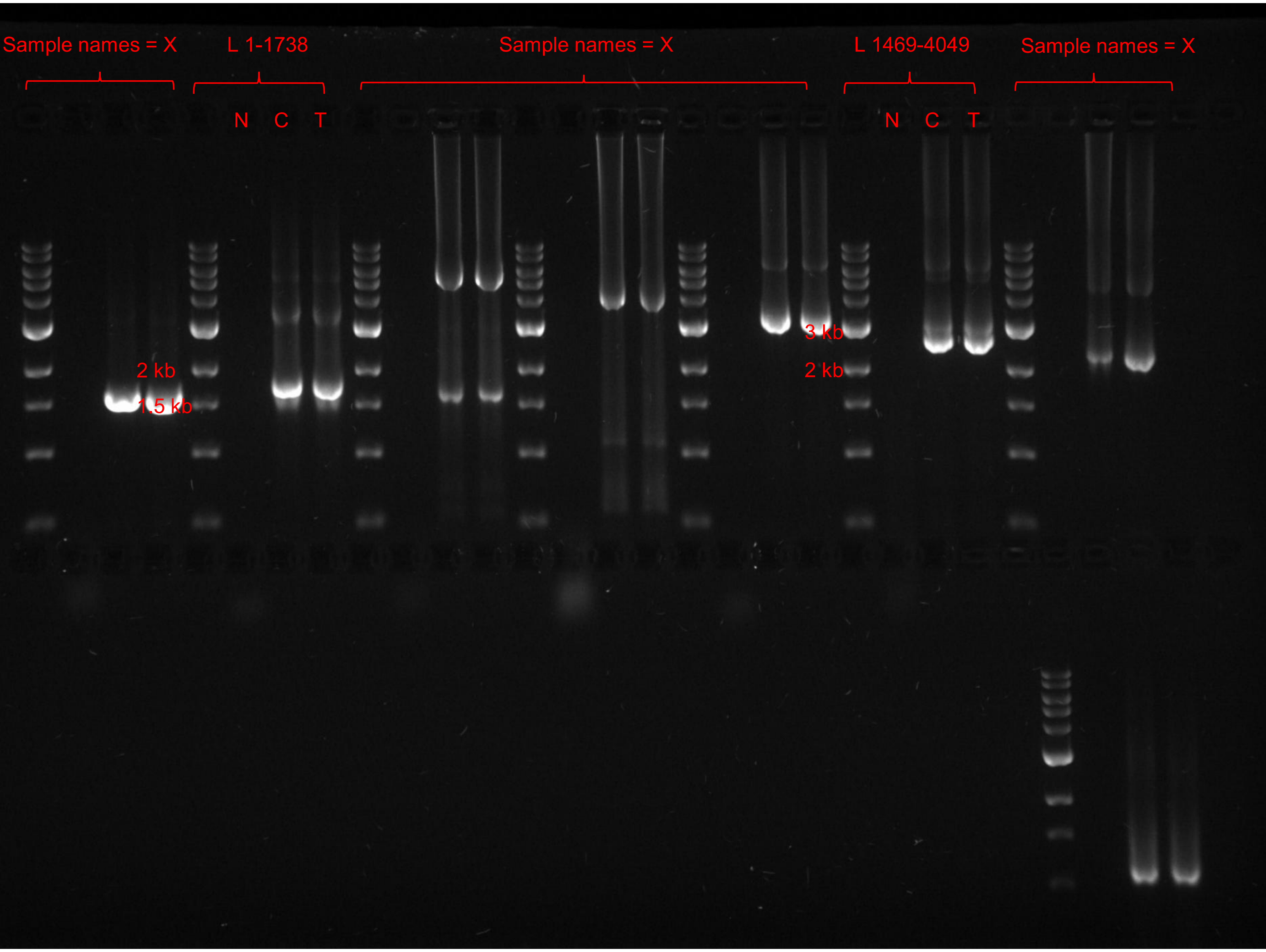

Raw agarose gel image for Fig 1D panels L 1-1738 and L 1469-4049. Lanes that were not included in the final figure are labelled with sample names “X”. Agarose gel image was acquired on a BioRad ChemiDoc Imaging System using the Image Lab Touch Software v3.0.1.14.

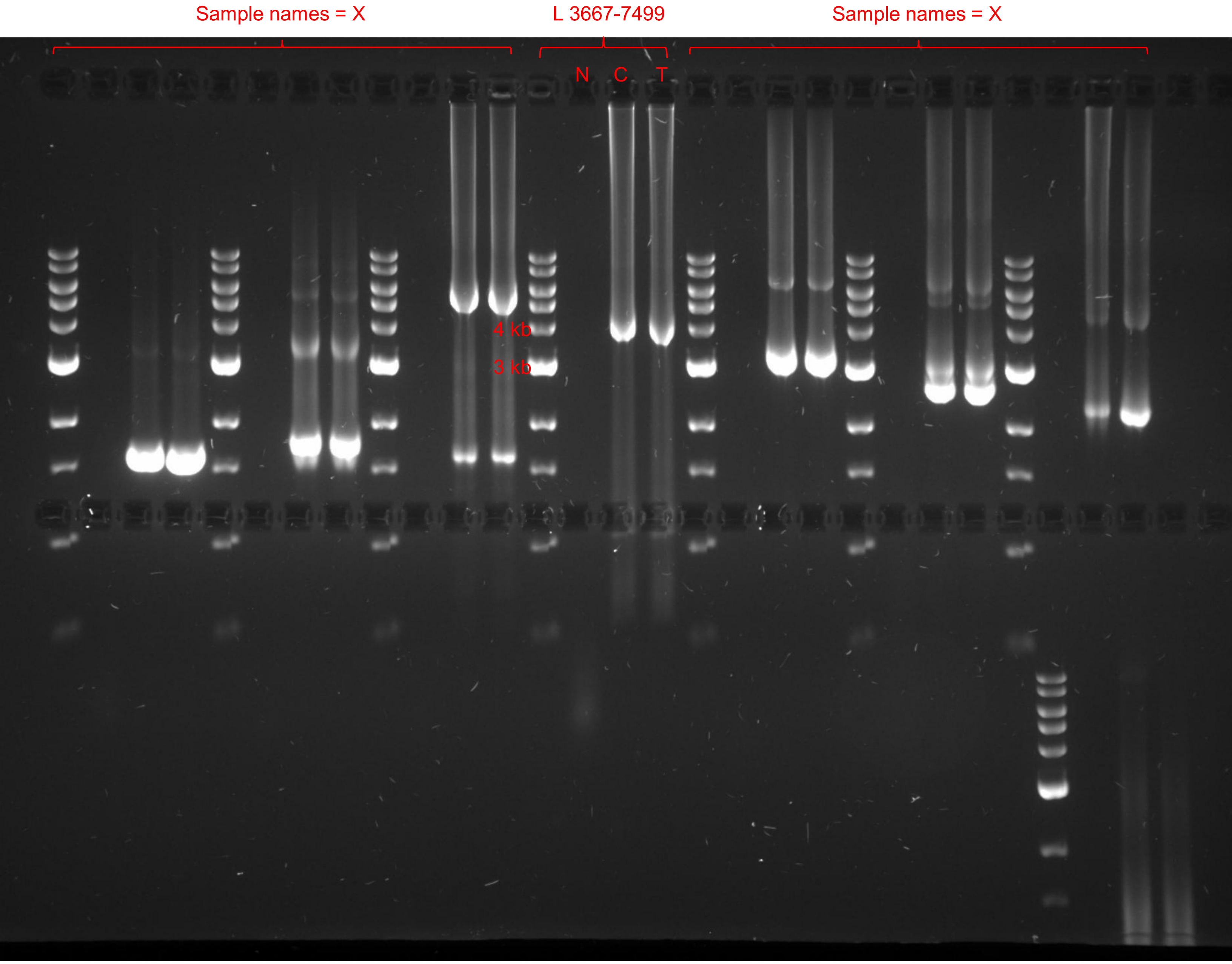

Raw agarose gel image for Fig 1D panel L 3667-7499. Lanes that were not included in the final figure are labelled with sample names “X”. Agarose gel image was acquired on a BioRad ChemiDoc Imaging System using the Image Lab Touch Software v3.0.1.14.

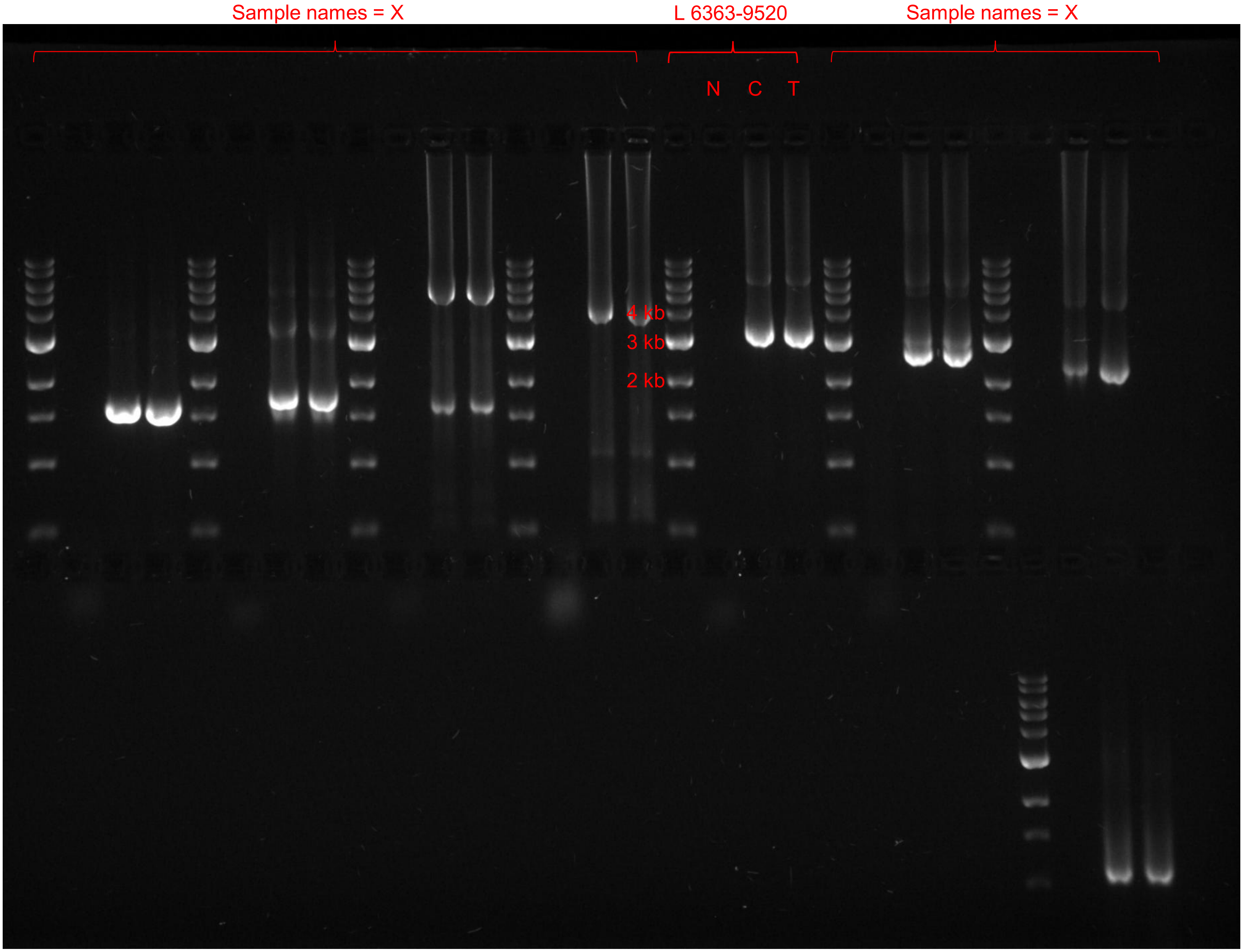

Raw agarose gel image for Fig 1D panel L 6363-9520. Lanes that were not included in the final figure are labelled with sample names “X”. Agarose gel image was acquired on a BioRad ChemiDoc Imaging System using the Image Lab Touch Software v3.0.1.14.

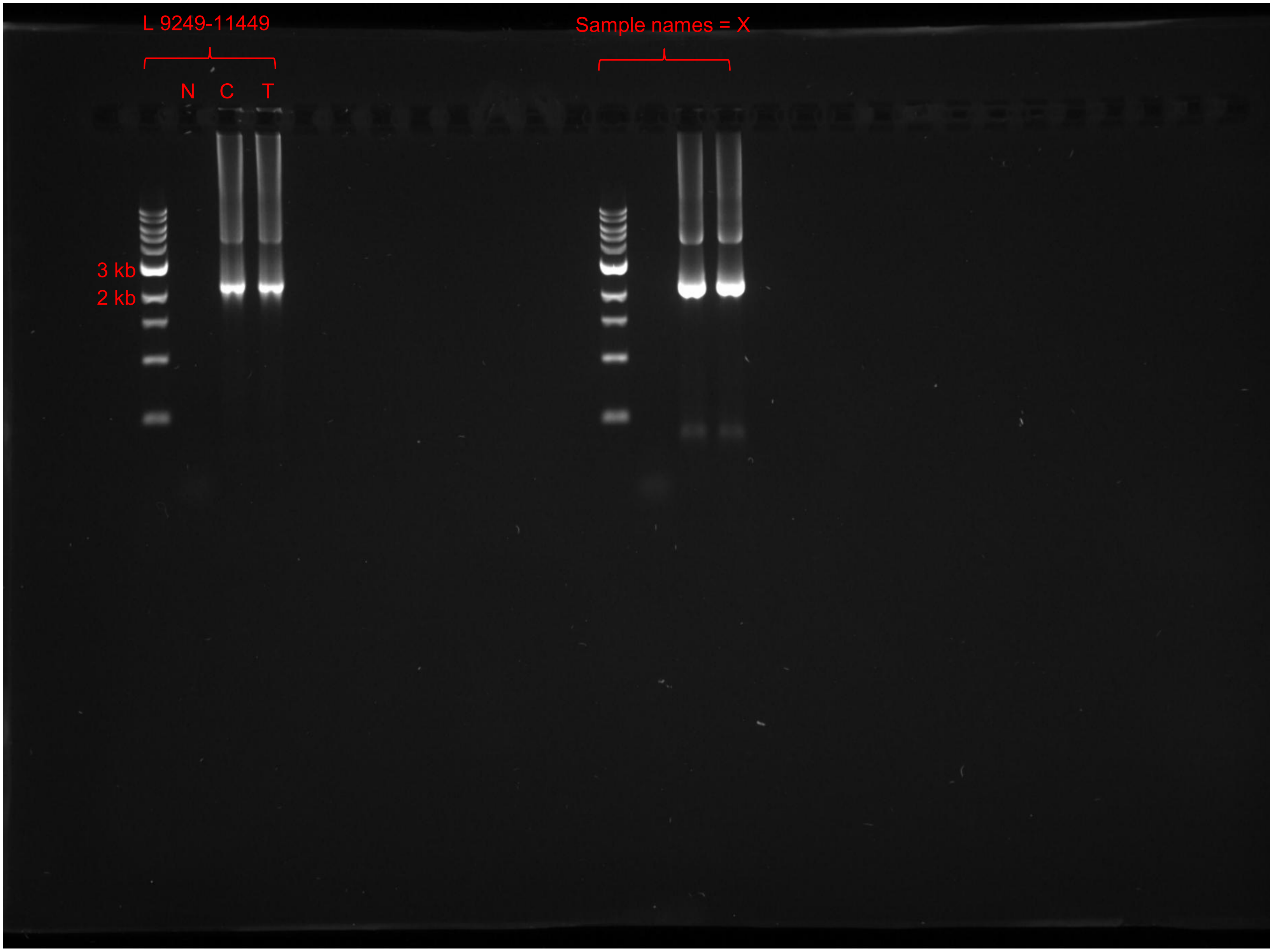

Raw agarose gel image for Fig 1D panel L 9249-11449. Lanes that were not included in the final figure are labelled with sample names “X”. Agarose gel image was acquired on a BioRad ChemiDoc Imaging System using the Image Lab Touch Software v3.0.1.14.

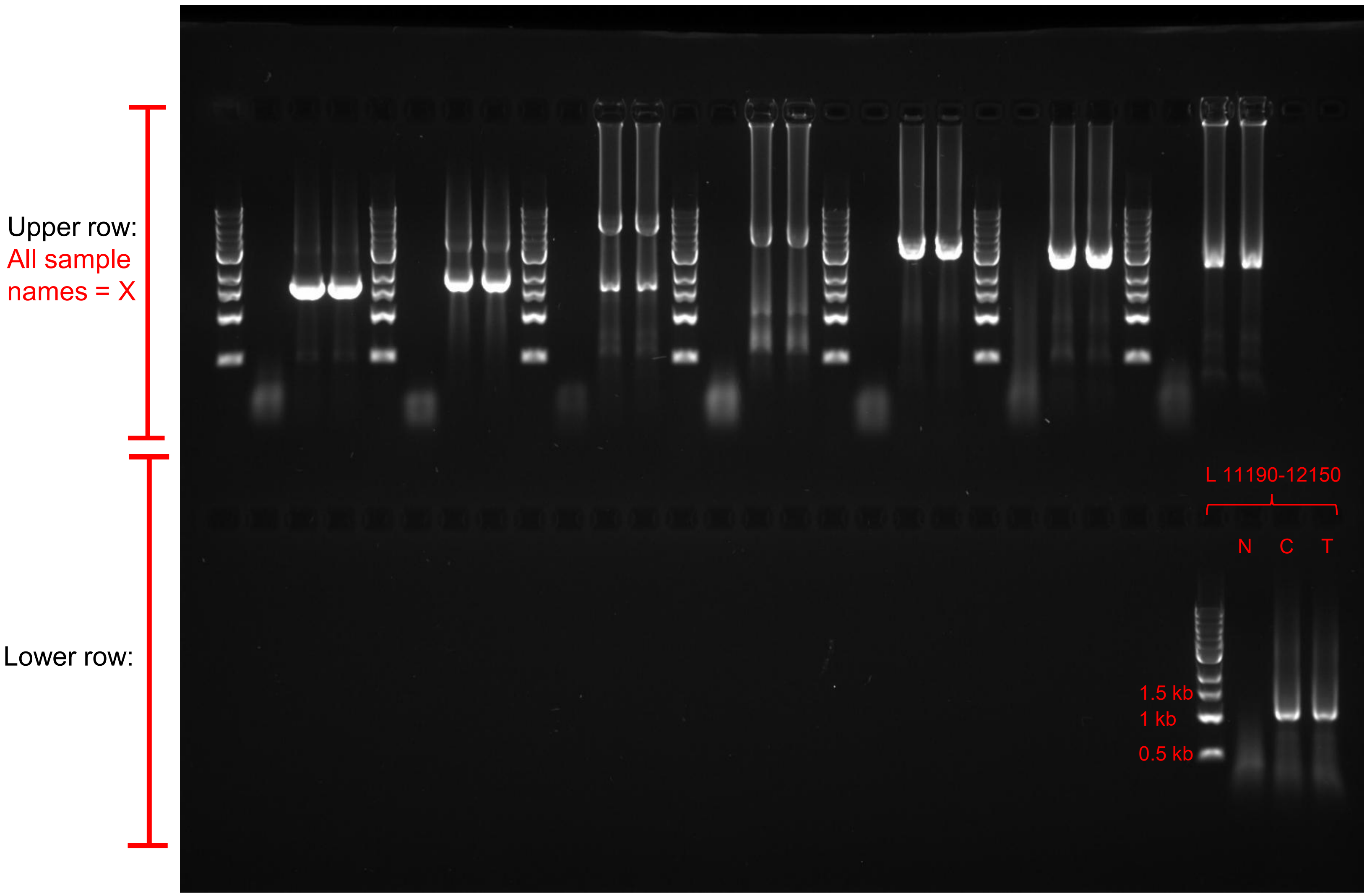

Raw agarose gel image for Fig 1D panel L 11190-12150. This gel had two rows and only the right part of the lower row was included in the final figure.. Upper-row Lanes, which were not included in the final figure, are assigned sample names “X”. Agarose gel image was acquired on a BioRad ChemiDoc Imaging System using the Image Lab Touch Software v3.0.1.14.

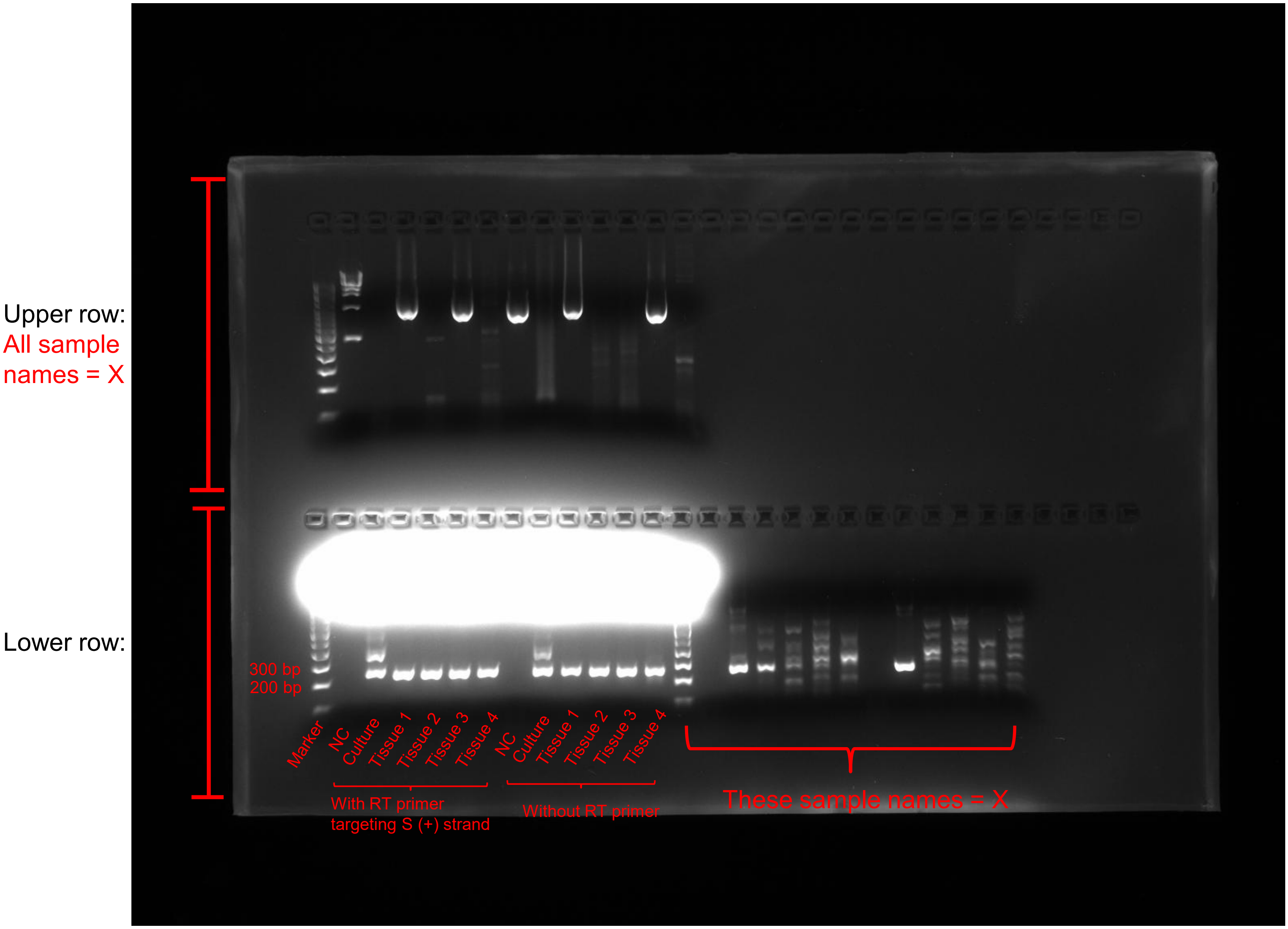

Raw agarose gel image for Fig F panel b in S1 File. Lanes that were not included in the final figure are assigned sample names “X”. This gel had two rows and only the left part of the lower row was included in the final figure. Electrophoresis was run with extended time to better separate the molecular size marker bands of 200 bp vs 300 bp. As a result, molecules of low molecular weights, likely excessive primers, from the samples of the upper row migrated into the lower part of the gel (the bright block of signals). This did not affect the detection of PCR products of interest, which fell between 200 bp and 300 bp. Agarose gel image was acquired on a BioRad ChemiDoc Imaging System using the Image Lab Touch Software v3.0.1.14.

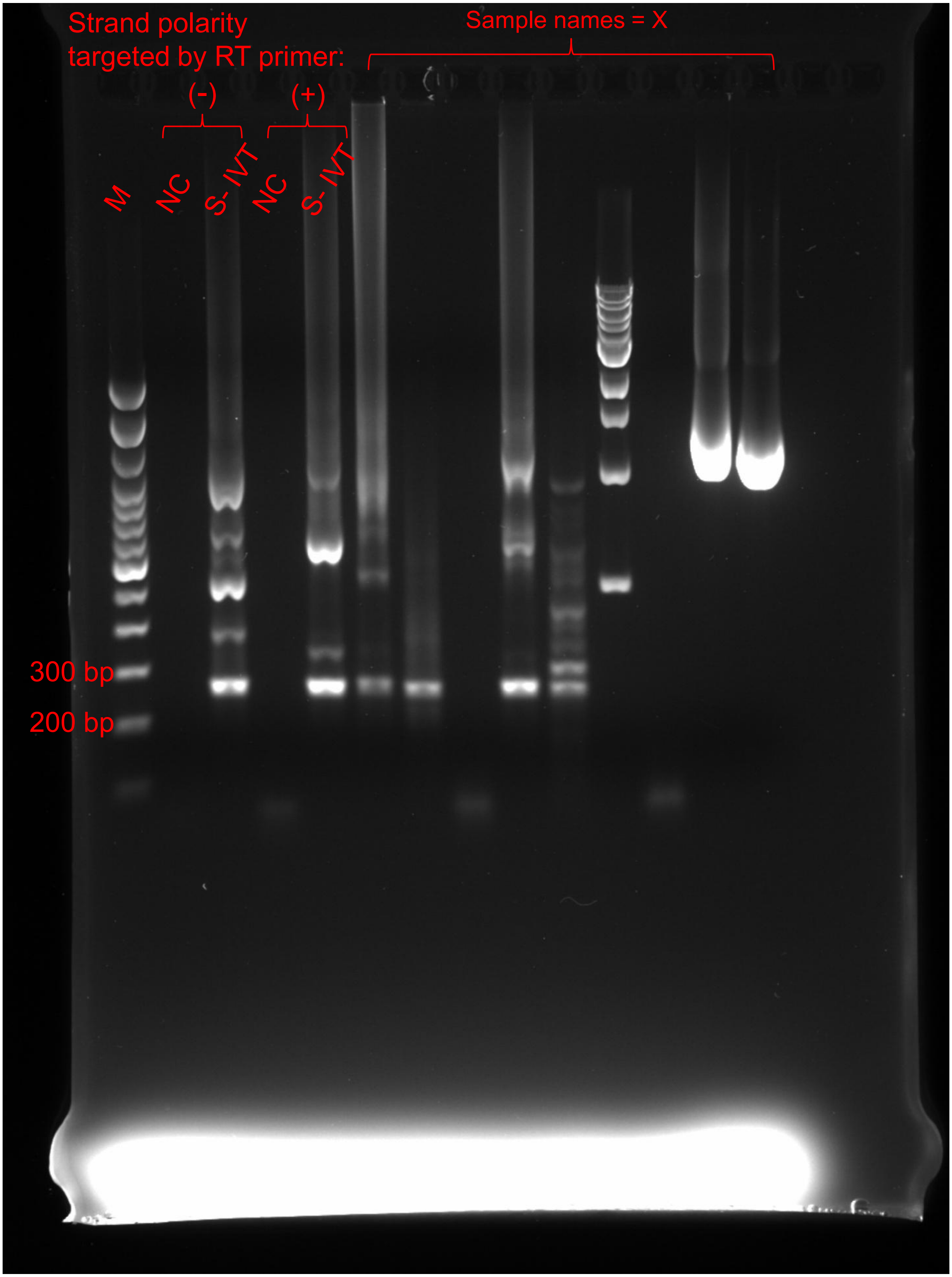

Raw agarose gel image for Fig F panel c in S1 File. Lanes that were not included in the final figure are labelled with sample names "X". Agarose gel image was acquired on a BioRad ChemiDoc Imaging System using the Image Lab Touch Software v3.0.1.14.

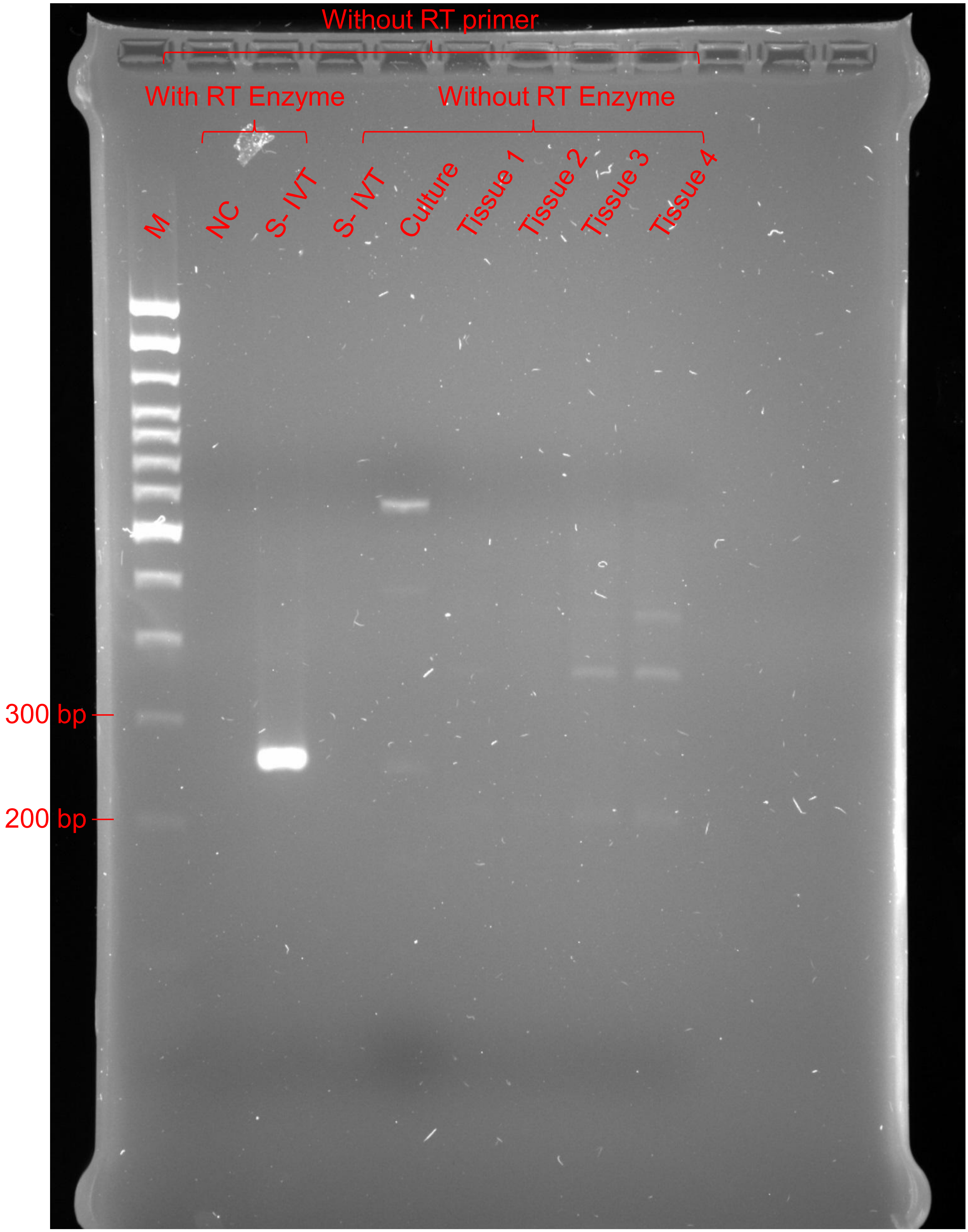

Raw agarose gel image for Fig F panel d in S1 File. Agarose gel image was acquired on a BioRad ChemiDoc Imaging System using the Image Lab Touch Software v3.0.1.14.

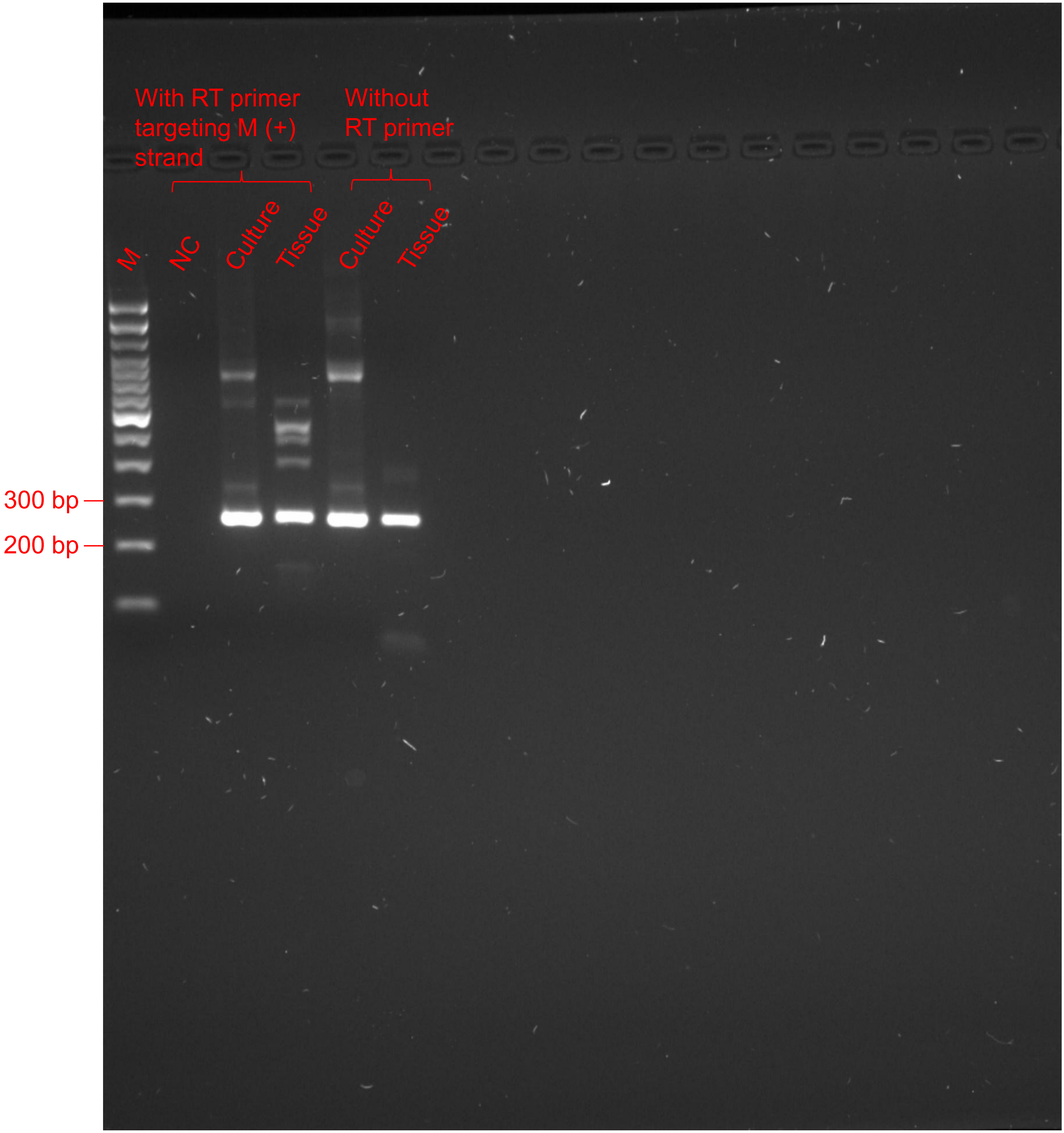

Raw agarose gel image for Fig F panel e in S1 File. Agarose gel image was acquired on a BioRad ChemiDoc Imaging System using the Image Lab Touch Software v3.0.1.14.

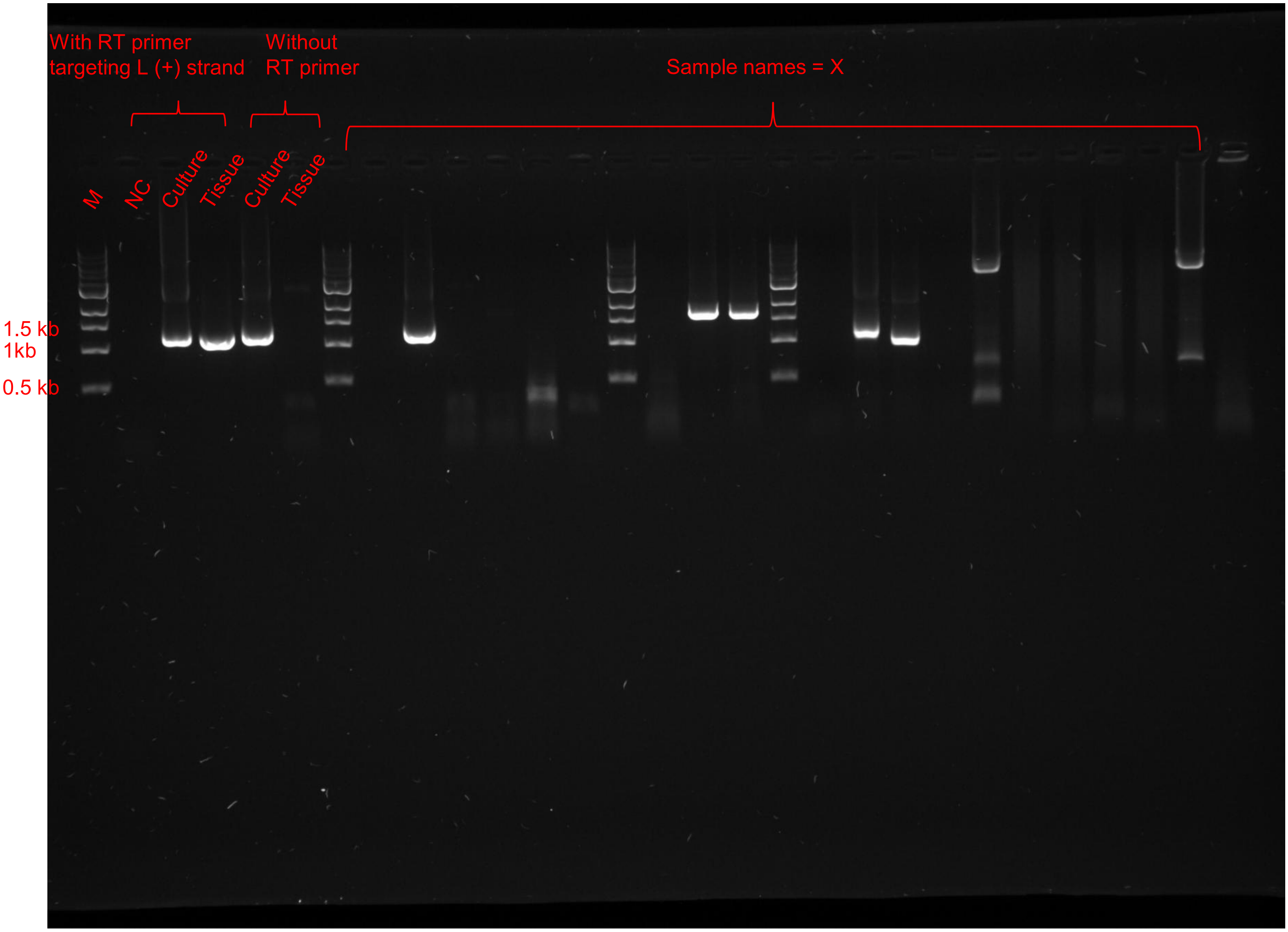

Raw agarose gel image for Fig F panel f in S1 File. Lanes that were not included in the final figure are labelled with sample names "X". Agarose gel image was acquired on a BioRad ChemiDoc Imaging System using the Image Lab Touch Software v3.0.1.14.

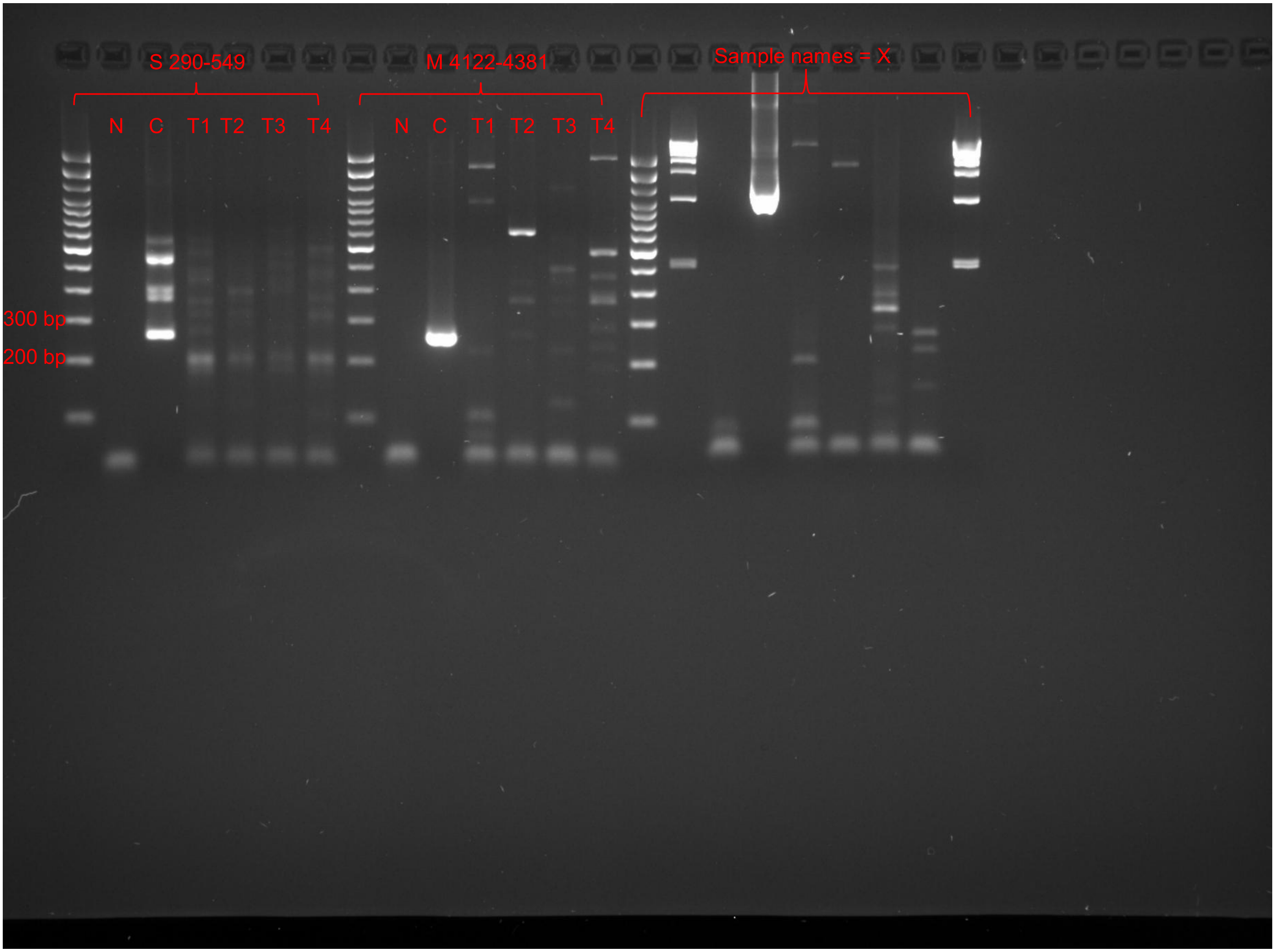

Raw agarose gel image for Fig G panel b subpanels S290-549 and M4122-4381 in S1 File. Lanes that were not included in the final figure are labelled with sample names "X". Agarose gel image was acquired on a BioRad ChemiDoc Imaging System using the Image Lab Touch Software v3.0.1.14.

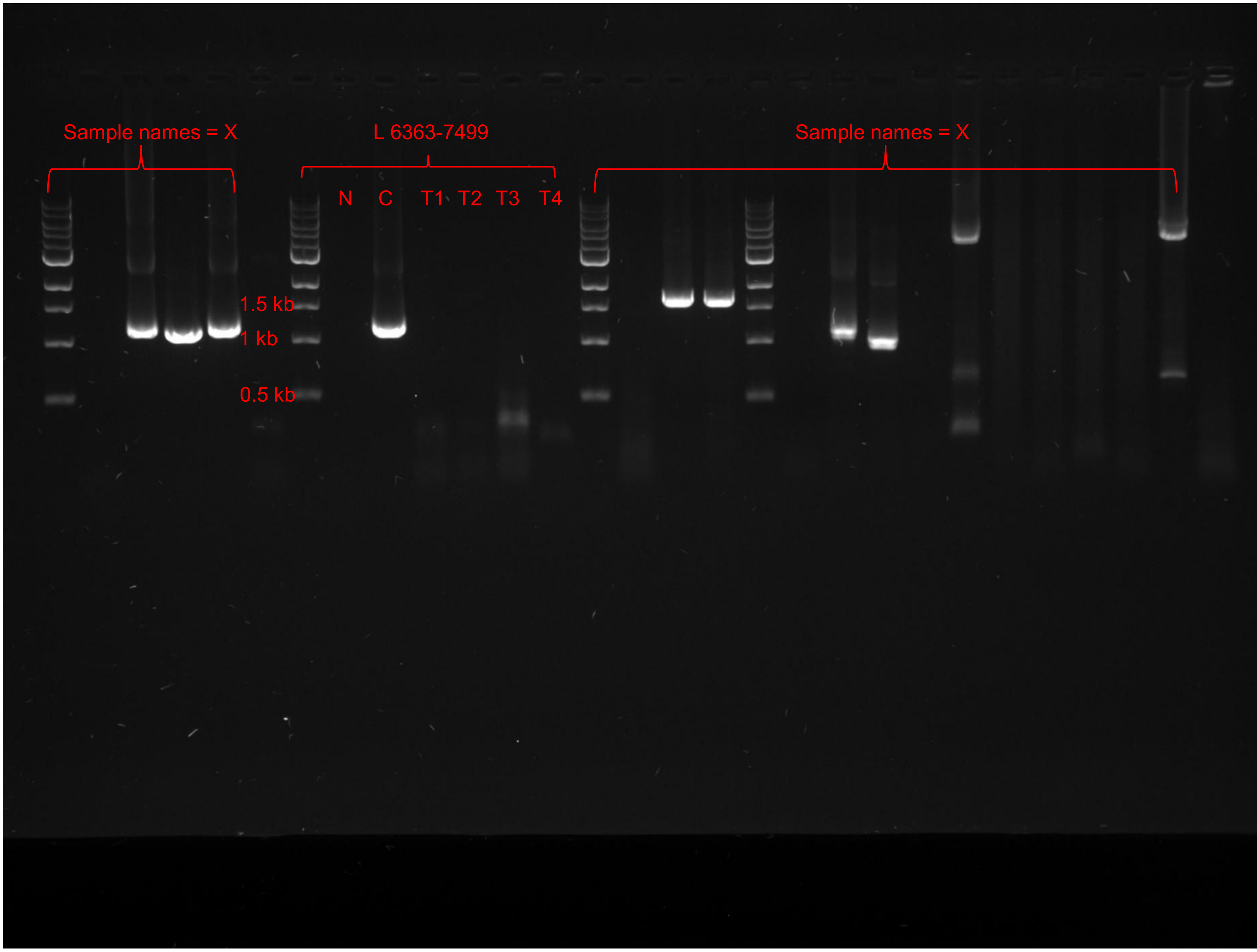

Raw agarose gel image for Fig G panel b subpanel L6363-7499 in S1 File. Lanes that were not included in the final figure are labelled with sample names "X". Agarose gel image was acquired on a BioRad ChemiDoc Imaging System using the Image Lab Touch Software v3.0.1.14.
