# Supplementary material for: Persistence of viral full-length genomic RNA and replication-associated RNA transcripts and proteins in sheep tissues following acute Crimean-Congo hemorrhagic fever virus infection
Source: PLoS Negl Trop Dis. 2026 Oct 30;20(10):e0014842. doi: 10.1371/journal.pntd.0014842 (PMC13734597; doi:10.1371/journal.pntd.0014842)
Supplement: Supplementary file 2 [file pntd.0014842.s001.docx]

**Supporting information**

**Persistence of viral full-length genomic RNA and replication-associated RNA transcripts and proteins in sheep tissues following acute Crimean-Congo hemorrhagic fever virus infection**

Hongzhao Li^1^, Estella Moffat^1^, Katherine Handel^1^, Michelle Nebroski^1^, Daniel Sullivan^1^, Andre Dufresne^1^, Carissa Embury-Hyatt^1^, Oliver Lung^1^ and Bradley S Pickering^1,2*^

^1^National Centre for Foreign Animal Disease, Canadian Food Inspection Agency, Winnipeg, Manitoba, Canada;

^2^Department of Medical Microbiology and Infectious Diseases, College of Medicine, Faculty of Health Sciences, University of Manitoba, Winnipeg, Manitoba, Canada

^*^ Correspondence to: bradley.pickering@inspection.gc.ca

**Table of contents**

Supplementary results and discussion …..………………..……...…………………………………………….....2

Supplementary methods …………………………………..……...…………………………………………….....4

Table A. RT-PCR conditions and primer sequences for amplifying CCHFV genomic RNA sequences .…….......6

Table B. Genomic mutations of CCHFV in sheep tissues .………………………..……..…………………….....7

Table C. Visualization of CCHFV RNA and protein in tissues .…………………..……..…………………….....8

Table D. RT-PCR conditions and primer sequences for targeting the strand polarity of CCHFV RNA transcripts .…………………………………………………………………………………………………..….....9

Fig A. Electron microscopy (EM) of a sheep lymph node tissue harboring persistent CCHFV RNA…………..10

Fig B. Image of ISH for negative-sense CCHFV RNA (M segment) displaying a larger area of tissue section ..11

Fig C. Image of ISH for positive-sense CCHFV RNA (M segment) displaying a larger area of tissue section ..12

Fig D. Image of IHC for CCHFV N protein displaying a larger area of tissue section……...…………………..13

Fig E. Hypothetical model of CCHFV RNA persistence in tissues ………..………………………….………...14

Fig F. RT-nested PCR targeting strand polarity of CCHFV RNA transcripts in total RNA extracts from sheep tissues …………………………………………………………………………...…………..…………………...15

Fig G. Detection of CCHFV sequences from pull-downs of poly(A)-containing RNA …………..…………….16

References …………………………………………………………………………..…………………………...17

**Supplementary results and discussion**

**Assessment of strand-specific RT-nested PCR targeting RNA transcripts indicative of viral replication**

We sought to examine positive-sense replicative intermediates (genomic cRNA and mRNA) in sheep tissues harboring persistent CCHFV RNA. To distinguish them from negative-sense genomic vRNA sequences in total tissue RNA extracts, we designed strand-specific RT-nested PCR methods (Fig F and Table D in S1 File). Following an RT reaction with a positive sense-targeting primer, RNA was digested, RT enzyme inactivated and the resulting cDNA affinity-purified prior to being used as the template for PCR, to prevent any non-specific RT reaction from being primed by PCR primers recognizing negative RNA strands. These methods generated positive detections of S segment sequences with cell culture and tissue-derived templates. Interestingly, however, results remained positive in control tests with no RT primer added. This suggests a false priming of the RT reaction independent of any primer addition and consequently impaired strand-specific detection. False priming was observed in previous assays intended to target the strand polarity of replicative intermediates in various viruses including Hantaan virus, a bunyavirus [1]. Consistent with this possibility, positive results were similarly produced using in vitro-transcribed negative-sense S segment RNA as the template in the presence of either an RT primer targeting the negative sense or an RT primer targeting the positive sense, or even in the absence of any RT primer. For all the positive detections without an RT primer, the possibility of DNA template contamination was excluded as they required the addition of RT enzyme. These results therefore confirm an impaired strand-specificity for the detection of positive-sense S segment transcripts. Similar findings were obtained for M segment transcripts with cell culture and tissue-derived templates and for L segment transcripts with cell culture-derived templates. Underlying mechanisms of false RT priming may be explained by the snapped-back priming model proposed previously for other viruses [1], or additionally by nonspecific priming by a cellular sequence in the context of total RNA extracts from tissues (Fig F in S1 File). Together, these data add to the literature for strand-specific detection of viral replicative intermediates by including CCHFV as a new target virus with lessons learned and nevertheless provide additional evidence for the presence of persisting CCHFV RNA sequences using alternative assays.

**Detection of CCHFV sequences in poly(A)-selected RNA pull-downs**

We investigated whether CCHFV mRNA was present in sheep tissues harboring persistent viral genomic RNA. Most eukaryotic mRNAs, as well as many viral mRNAs, possess a poly(A) tail [2], which enables affinity purification via oligo(dT) pull-down methods [3, 4]. Although bunyavirus mRNAs are generally considered non-polyadenylated [5], hantavirus M-segment mRNAs have been reported to acquire poly(A) tails using a poly(U) transcription termination signal [6]. Concerning CCHFV, the 3′-terminal processing of its mRNAs has not been well characterized [7].

Using oligo(dT)-based enrichment, we did not detect CCHFV poly(A)-containing RNA in sheep tissues collected at 34 DPI. In contrast, parallel pull-downs from infected cell cultures yielded detectable CCHFV sequences corresponding to all three genomic segments (S, M and L) (Fig G in S1 File). Taken at face value, these findings might suggest that polyadenylated CCHFV RNA is present during active replication in cell culture but absent in persistently infected sheep tissues.

However, a more cautious interpretation is warranted: Re-examination of CCHFV genomic sequences did not reveal canonical polyadenylation signals, including the poly(U) polyadenylation motif described for hantaviruses [2, 6]. Together with the prevailing evidence that bunyavirus mRNAs are typically non-polyadenylated [5], these observations argue against the existence of bona fide poly(A) tails on CCHFV transcripts. Instead, the detection of CCHFV sequences in oligo(dT) pull-downs from the viral RNA-rich cell culture extracts is more plausibly explained by indirect or artifactual mechanisms. These may include co-purification of viral RNA with host poly(A) RNAs within ribonucleoprotein complexes [3, 4], or nonspecific retention and carryover of viral RNA due to incomplete washing or bead-associated background binding. Such effects would be expected to be more pronounced in samples with high viral RNA abundance, such as actively infected cell cultures.

**Study limitation**

It should be noted that findings in this study were obtained from experimental infection through subcutaneous and intravenous injections in sheep of young age [8]. While using a clinically relevant CCHFV isolate associated with high pathogenicity in humans (Kosovo Hoti), the inoculation route might not faithfully recapitulate the course of natural infection through tick bites and sheep at the young age (approximately two months old) are immunologically immature compared with adult sheep [9, 10]. Future studies should compare the effects of infection route, dose, viral strain and animal age on long-term outcomes.

**Supplementary methods**

**Electron microscopy (EM)**

EM was performed on the Sheep 4 inguinal lymph node. This lymph node was positive for both viral RNA (negative-sense and positive-sense) and protein in tissue staining experiments (Table C in S1 File) and was thus selected as the most promising target for EM investigation. Tissue samples were processed for conventional sample preparation (fixing, embedding, sectioning and staining of tissue) and examined for the presence of virus-like particles. Tissues received in 10% buffered formalin were fixed for two hours in 2.5% glutaraldehyde and 2% paraformaldehyde in 0.1 M sodium phosphate buffer (pH 7.2) at 4°C. After washing in the buffer, tissues were post-fixed in 1% osmium tetroxide for one hour. Samples were dehydrated using an ethanol gradient ending in 100% propylene oxide. Samples were infiltrated and embedded in epoxy resin (araldite 505/embed-812). 70-nm sections were obtained using a Leica Ultracut UCT and collected on 200 hexagon mesh copper grids. Contrast staining was accomplished with freshly prepared aqueous 2% uranyl acetate and fresh lead citrate stains. Observations were made using a Delong Instrument LVEM 25E in TEM (25KV) and STEM (15KV) mode of operation. Two samples from two different areas of the lymph node were processed to generate two blocks of embedded tissue for each area, respectively. One block from each tissue sample was sectioned and stained for observation. 10 grids of each block were sectioned for each block, and four grids of each sample were examined over a period of several days to complete the project.

**RT-nested PCR targeting the strand polarity of CCHFV RNA transcripts**

Strand polarity-specific RT-nested PCR strategy is outlined in Fig F in S1 File. RT reactions were each based on an RT primer designed to recognize a target viral RNA sequence of a specific strand polarity. Regarding the common reaction formulations and conditions, RT reactions were performed using the Maxima H Minus First Strand cDNA Synthesis Kit (ThermoFisher Scientific, K1652) as described in the main methods, with the following exceptions: The Maxima H Minus Enzyme Mix (containing RNase inhibitor RiboLock) was replaced by Maxima H Minus Reverse Transcriptase (ThermoFisher Scientific, EP0752, containing no RNase inhibitor); only a 100 µM RT primer master stock was used; and two RT reaction temperatures were tested: 50°C and 65°C. PCR reactions were performed using the LongAmp Taq PCR method as described in the main methods. Amplicon-specific variations in reaction conditions and primer sequences are detailed in Table D in S1 File. To remove RNA and RT enzyme and purify cDNA, 20 µl RT reaction was digested with 1 µl RNase H (New England BioLabs, M0297L) and 1 µl RNase A from the RNaseAlert Lab Test Kit v2 (ThermoFisher Scientific, 4479768) at 37 °C for one hour and then at room temperature overnight, followed by purification with MinElute Gel Extraction Kit (Qiagen, 28604).

**Detection of CCHFV sequences from pull-downs of poly(A)-containing RNA**

Poly(A)-containing RNA was purified from total RNA extracts from tissue homogenates with Dynabead mRNA Purification Kit (ThermoFisher Scientific, 61006) by following the supplier’s protocol [11] with these customizations: 40 µl input RNA was combined with 60 µl 10 mM Tris-HCl, pH 7.5 to serve as the starting material. mRNA bound to the beads was finally eluted in two rounds: first round with 20 µl Tris-HCl, heated at 78°C for 2 minutes; second round with 5 µl, heated at 80°C for approximately 4 minutes. Each heating step was immediately followed by placing the tube on the magnet for collection of the mRNA elute separated from the beads. The elutes from both rounds were finally combined and analyzed by positive sense-targeting RT-nested PCR described above.

**Production of the S segment RNA genome by in vitro transcription with enhanced DNA removal (S-IVT)**

The negative-sense S segment RNA genome of CCHFV Kosovo Hoti was in vitro-transcribed, treated with DNase I and column-purified as previously described [12]. This was diluted at 1:200 in H2O and further treated with the DNA-free DNA Removal Kit (ThermoFisher Scientific, AM1906), in a digestion reaction consisting of 83 µl H2O, 5 µl diluted RNA, 10 µl 10× Reaction Buffer and 2 µl rDNase with incubation at 37 °C for 30 minutes. The digestion reaction was then cleaned up using the RNeasy Mini Kit (Qiagen, 74104) with RNA elution in 30 µl H2O. The eluted RNA was further treated with restriction enzyme digestion in a reaction containing 22 µl H2O, 20 µl RNA, 5 µl 10× NEBuffer EcoRI/SspI and 3 µl EcoRI (New England Biolabs, R0101S) with incubation at 37 °C for one hour. The digestion reaction was finally cleaned up using the RNeasy Mini Kit with RNA elution in 30 µl Core elution buffer from the MagMAX CORE Nucleic Acid Purification Kit (ThermoFisher Scientific, A32700).

**Fig A. Electron microscopy (EM) of a sheep lymph node tissue harboring persistent CCHFV RNA.** EM was performed on the Sheep 4 inguinal lymph node. Representative images are shown. **a.** Image with a broader field encompassing multiple cells. **b.** Image at a higher magnification focused on the cell in the lower left area of panel a. The red annotation in the cytoplasm denotes a reference distance of approximately 100 nm. Virions of CCHFV and other bunyaviruses are known to be spherical particles in the cytoplasm with a diameter of approximately 100 nm and an envelope structure [13-15]. No virus-like particles exhibiting these characteristic ultrastructural features were observed.

.

**Fig B. Image of ISH for negative-sense CCHFV RNA (M segment) displaying a larger area of tissue section.** Arrows point to areas of positive staining.

**Fig C. Image of ISH for positive-sense CCHFV RNA (M segment) displaying a larger area of tissue section.** Arrow points to area of positive staining.

**Fig D. Image of IHC for CCHFV N protein displaying a larger area of tissue section.** Arrow points to area of positive staining.

**Fig E. Hypothetical model of CCHFV RNA persistence in tissues.** Genomic RNAs may be maintained as ribonucleoprotein complexes (RNPs) with the N and RdRp proteins. Sporadic reactivation of viral gene expression or replication could occur in response to immune control relaxation and external stimuli. This could contribute to the maintenance or expansion of persistent RNA and protein. Viral RNA persistence could originate through two possible routes, depending on the type and antiviral state of the host cell. During a recent viral replication, the host cell may proceed to a relatively quiescent state in which active virion production becomes inhibited while genomic RNA is maintained. Alternatively, an infected host cell may directly enter a quiescent state harboring persistent genomic RNA, without virion production. Genomic vRNA and cRNA may remain in a static state in long-lived cells or replicate using each other as a template and be passed on to new cells through cell division or cell-to-cell transfer of viral RNPs. It is important to note that viral gene expression (RNA transcription and protein translation) may not necessarily result in the release of whole virions. Whether a reactivation of the persistent viral RNA reservoir leads to the production of infectious virus remains unknown but represents a key future direction of investigation.

**Fig F. RT-nested PCR targeting strand polarity of CCHFV RNA transcripts in total RNA extracts from sheep tissues.** **a.** Strand-specific RT-nested PCR procedure exemplified for targeting positive-sense transcripts. Red and green colors represent positive sense (+) and negative sense (-) of CCHFV sequences, respectively. **b.** Agarose gel of PCR products from RT-nested PCR for detecting S segment transcripts, in the presence or absence of an RT primer aimed at the positive sense. The same results were obtained using an RT temperature of either 50 °C or 65 °C in these experiments. A common RT temperature of 50 °C was used in all other experiments. **c.** RT-nested PCR using an RT primer aimed at an S segment sequence of either negative or positive sense. S-IVT, S segment genomic RNA produced by in vitro transcription, of negative sense only. **d.** RT-nested PCR without RT primer, and with or without RT enzyme, for detecting S segment transcripts. **e and f.** RT-nested PCR for detecting M or L segment transcripts, in the presence or absence of an RT primer aimed at the positive sense. **g.** Hypothetical mechanisms for impaired strand-specific detection.

**Fig G. Detection of CCHFV sequences from pull-downs of poly(A)-containing RNA.** **a.** Poly(A)-containing RNA was pulled down using magnetic oligo(dT) beads, while other components from total tissue RNA extracts were washed off. The purified RNA was then analyzed by RT-nested PCR. **b.** Agarose gel images. N, negative control without viral template. C, CCHFV cell culture. T1 – T4, lymph nodes from different sheep. PCR product bands from cell culture were confirmed by sequencing to be of expected CCHFV sequences.

**References**

1.	Tuiskunen A, Leparc-Goffart I, Boubis L, Monteil V, Klingstrom J, Tolou HJ, et al. Self-priming of reverse transcriptase impairs strand-specific detection of dengue virus RNA. J Gen Virol. 2010;91(Pt 4):1019-27. Epub 20091125. doi: 10.1099/vir.0.016667-0. PubMed PMID: 19940062.

2.	Cross ST, Michalski D, Miller MR, Wilusz J. RNA regulatory processes in RNA virus biology. Wiley Interdiscip Rev RNA. 2019;10(5):e1536. Epub 20190429. doi: 10.1002/wrna.1536. PubMed PMID: 31034160; PubMed Central PMCID: PMCPMC6697219.

3.	Baltz AG, Munschauer M, Schwanhausser B, Vasile A, Murakawa Y, Schueler M, et al. The mRNA-bound proteome and its global occupancy profile on protein-coding transcripts. Mol Cell. 2012;46(5):674-90. doi: 10.1016/j.molcel.2012.05.021. PubMed PMID: 22681889.

4.	Matia-Gonzalez AM, Iadevaia V, Gerber AP. A versatile tandem RNA isolation procedure to capture in vivo formed mRNA-protein complexes. Methods. 2017;118-119:93-100. Epub 20161013. doi: 10.1016/j.ymeth.2016.10.005. PubMed PMID: 27746303.

5.	Malet H, Williams HM, Cusack S, Rosenthal M. The mechanism of genome replication and transcription in bunyaviruses. PLoS Pathog. 2023;19(1):e1011060. Epub 20230112. doi: 10.1371/journal.ppat.1011060. PubMed PMID: 36634042; PubMed Central PMCID: PMCPMC9836281.

6.	Hutchinson KL, Peters CJ, Nichol ST. Sin Nombre virus mRNA synthesis. Virology. 1996;224(1):139-49. doi: 10.1006/viro.1996.0515. PubMed PMID: 8862408.

7.	Zivcec M, Scholte FE, Spiropoulou CF, Spengler JR, Bergeron E. Molecular Insights into Crimean-Congo Hemorrhagic Fever Virus. Viruses. 2016;8(4):106. Epub 20160421. doi: 10.3390/v8040106. PubMed PMID: 27110812; PubMed Central PMCID: PMCPMC4848600.

8.	Li H, Pinette M, Smith G, Goolia M, Handel K, Nebroski M, et al. Distinguishing host responses, extensive viral dissemination and long-term viral RNA persistence in domestic sheep experimentally infected with Crimean-Congo haemorrhagic fever virus Kosovo Hoti. Emerg Microbes Infect. 2024;13(1):2302103. Epub 20240122. doi: 10.1080/22221751.2024.2302103. PubMed PMID: 38189080; PubMed Central PMCID: PMCPMC10810640.

9.	Nguyen TC. The immune response in sheep: analysis of age, sex and genetic effects on the quantitative antibody response to chicken red blood cells. Vet Immunol Immunopathol. 1984;5(3):237-45. doi: 10.1016/0165-2427(84)90037-0. PubMed PMID: 6719821.

10.	Watson DL, Colditz IG, Andrew M, Gill HS, Altmann KG. Age-dependent immune response in Merino sheep. Res Vet Sci. 1994;57(2):152-8. doi: 10.1016/0034-5288(94)90051-5. PubMed PMID: 7817003.

11.	ThermoFisher. Dynabeads mRNA Purification Kit. Available from: <https://documents.thermofisher.com/TFS-Assets/LSG/manuals/MAN0015808_Dynabeads_mRNA_Purification_UG.pdf> [Cited 2026 February 13]. Thermo Fisher Scientific. 2016.

12.	Li H, Bello A, Smith G, Kielich DMS, Strong JE, Pickering BS. Degenerate sequence-based CRISPR diagnostic for Crimean-Congo hemorrhagic fever virus. PLoS Negl Trop Dis. 2022;16(3):e0010285. Epub 2022/03/11. doi: 10.1371/journal.pntd.0010285. PubMed PMID: 35271569; PubMed Central PMCID: PMCPMC8939784.

13.	Xia H, Zhao J, Li Y, Yin S, Tang S, Zhang Z, et al. Infection and propagation of Crimean-Congo hemorrhagic fever virus in embryonated chicken eggs. Virus Res. 2013;173(2):344-9. Epub 20130123. doi: 10.1016/j.virusres.2013.01.008. PubMed PMID: 23352881.

14.	Kuhn JH, Alkhovsky SV, Avsic-Zupanc T, Bergeron E, Burt F, Ergunay K, et al. ICTV Virus Taxonomy Profile: Nairoviridae 2024. J Gen Virol. 2024;105(4). doi: 10.1099/jgv.0.001974. PubMed PMID: 38687001; PubMed Central PMCID: PMCPMC11094370.

15.	Overby AK, Pettersson RF, Grunewald K, Huiskonen JT. Insights into bunyavirus architecture from electron cryotomography of Uukuniemi virus. Proc Natl Acad Sci U S A. 2008;105(7):2375-9. Epub 20080212. doi: 10.1073/pnas.0708738105. PubMed PMID: 18272496; PubMed Central PMCID: PMCPMC2268144.
