# Supplementary material for: Persistence of viral full-length genomic RNA and replication-associated RNA transcripts and proteins in sheep tissues following acute Crimean-Congo hemorrhagic fever virus infection
Source: PLoS Negl Trop Dis. 2026 Oct 30;20(10):e0014842. doi: 10.1371/journal.pntd.0014842 (PMC13734597; doi:10.1371/journal.pntd.0014842)
Supplement: Supplementary file 3 [file pntd.0014842.s003.docx]

**Response to Reviewers**

**Reviewers' Comments:**

Reviewer's Responses to Questions

**Key Review Criteria Required for Acceptance?**

As you describe the new analyses required for acceptance, please consider the following:

**Methods**
-Are the objectives of the study clearly articulated with a clear testable hypothesis stated?
-Is the study design appropriate to address the stated objectives?
-Is the population clearly described and appropriate for the hypothesis being tested?
-Is the sample size sufficient to ensure adequate power to address the hypothesis being tested?
-Were correct statistical analysis used to support conclusions?
-Are there concerns about ethical or regulatory requirements being met?

**Reviewer #1:** Please see "Summary and General Comments".

**Reviewer #2:** The authors could make it clearer that this paper investigated the characteristics of CCHFV RNA in lymphoid tissues from which complete genome sequence was previously obtained. This is mentioned in the results but would be useful to include in the objectives. Similarly that there were technical difficulties previously that the researchers used several methods overcome.

**Response:**

We thank the reviewer for these helpful suggestions, which have improved the clarity and contextualization of the manuscript. In response, we have revised both the Introduction and Results sections to more clearly state that this study investigated the characteristics of CCHFV RNA in lymphoid tissues from which partial viral genome sequences had previously been detected. We have also highlighted the technical challenges encountered in the earlier work and described the new methodological approaches developed in the present study to overcome these limitations.

CCHFV persistence was investigated using lymphoid tissues collected from our previous experimental infection study, in which partial sequences of the S segment remained detectable several weeks after the acute viremia had ended [Li et al. 2024. PMID 38189080]. However, due to technical challenges, our earlier efforts during that original experimental infection study were unable to conclusively determine whether full-length CCHFV genomic RNA and molecular markers of viral replication persisted in post-acute sheep tissues. In the present study, we revisited archived tissue samples from that prior infection study using newly developed approaches and obtained evidence supporting the long-term persistence of viral whole genome RNA sequences, accompanied by the presence of replication-associated viral transcripts and proteins.

The related revisions have been incorporated in the Introduction and Results sections of the revised manuscript (Lines 83 – 90, 106 – 112, 126 – 132 and 157 – 168).

The authors refer to a related paper for the experimental infection of the sheep. It was also not clear if the samples were obtained from the previous experiment although I assumed they were due to the similarity of sampling intervals etc. I understand the authors do not want to repeat the entire methodology however I strongly suggest that a summary of the sheep experiments be included. It was not possible to evaluate this paper without accessing the previous publication. The authors presented evidence that viral whole genome RNA sequences persist in tissues after acute viremia, and detected replication associated viral RNA transcripts and viral proteins. The authors could briefly provide the experimental approach to assist with understanding the results presented in this paper. Where did the primers come from? There is an extensive table, were these all designed in house and what strategy?

**Response:**

We thank the reviewer for this valuable comment. The reviewer is correct that the samples analyzed in the present study were obtained from the sheep experimental infection study reported previously. We agree that a concise summary of that study is important for providing context and allowing readers to understand the present work without having to consult the earlier publication. Accordingly, we have included a summary of the sheep infection study (Lines 119 – 132) as well as a schematic overview (Fig 1A).

To further facilitate interpretation of the results presented in this study, we have also included a brief description and graphical illustration of the overall experimental approach used to recover and characterize viral genomic RNA sequences (Fig 1B and C, and accompanying figure legends). These provide readers with an overview of the workflow without requiring detailed examination of the methodological sections.

Regarding the RT-PCR assays, the primers, reaction formulations and amplification conditions were designed and optimized in-house. The overall approach was based on a nested-like PCR strategy involving repeated rounds of amplification to maximize analytical sensitivity. Since individual genomic regions differed in amplification performance, a variety of amplicon-specific primer designs and reaction conditions were developed and optimized. In the revised manuscript, the general RT-PCR procedure has been described in the Methods section (Lines 262 – 318) with the nested-like repeated PCR strategy illustrated in Fig 1B and C (Lines 140 – 150), and the detailed amplicon-specific primer sets and reaction conditions have been organized into Table A (Page 7) in S1 File (the Supporting information file).

We hope these revisions provide sufficient experimental context and clarify both the origin of the samples and the rationale underlying the primer design and amplification strategy leading to the recovery of persistent CCHFV genomic RNA sequences.

The methods describe the various RT-nested PCR reactions to amplify viral RNA, genomic RNA and targeting strand polarity of CCHFV RNA, perhaps these could be summarised and presently more succinctly using tables.

**Response:**

We thank the reviewer for this helpful suggestion and agree that the original description of the RT-PCR methods contained a large amount of technical details. To improve readability and present the methodology more concisely, we have revised the manuscript as follows: (1) The RT-PCR methods targeting strand polarity are now considered supplementary in nature in the updated manuscript. The corresponding methodological details have been moved to the Supplementary methods (Page 5) and Table D (Page 10) in S1 File. (2) In the main manuscript the description of RT-PCR methods has been restricted to the core methods used for recovery of viral genomic RNA sequences (Lines 262 – 318). (3) Since the most extensive methodological information concerns amplicon-specific variations in RT-PCR assays used to recover viral genomic RNA sequences, these details have been organized into a table. In accordance with the journal's editorial requirements, this large table has been included in the Supporting information as Table A (Page 7) in S1 File. (4) While streamlining the presentation, we have retained methodological details that we consider essential for reproducibility. We believe these details will be valuable to researchers seeking to apply or adapt the approaches developed in this study for future investigations of CCHFV persistence. We hope that these revisions improve the clarity and accessibility of the Methods section while preserving the information necessary for reproducibility.

Reviewer #3: See my comments #1 and #2.

**Results**


-Does the analysis presented match the analysis plan?

-Are the results clearly and completely presented?

-Are the figures (Tables, Images) of sufficient quality for clarity?

Reviewer #1: Please see "Summary and General Comments".

Reviewer #2: It is unclear exactly what tissues were positive and what tissues were homogenised for RNA extraction. A brief summary is needed. Moving some of the comments in results to methodology may assist.

The authors may want to consider removing "comments" from the figure legends and keep to a precise description of the figure.
The sequence data may be more suitable in supplementary data with a summary of the mutations
Fig 7. Were the tissues from day 34?
From how many sheep and tissues was CCHF sequence detected?

**Response:**

We thank the reviewer for these helpful suggestions, which have improved the clarity of the manuscript and the presentation of the data.

To clarify the tissues examined, four tissue samples that were positive for persistent CCHFV RNA were homogenized for RNA extraction and RT-PCR analysis. These included Sheep 1 deep cervical lymph nodes (LN), Sheep 2 inguinal LN, Sheep 3 gastrohepatic LN and Sheep 4 inguinal LN. This information has been added to the revised manuscript including in the methodology section (Lines 150 – 152 and 265 – 268).

We have also revised the figure legends by removing interpretive comments and limiting the legends to concise descriptions of the figures.

As suggested, the sequence data have been organized into a supplementary table with a summary of the mutations and presented as Table B (Page 8) in S1 File.

Regarding Fig 7 in the original manuscript, this figure has been incorporated into the new Fig 2, which combines in situ hybridization (ISH) for negative-sense viral RNA, ISH for positive-sense viral RNA, and immunohistochemistry (IHC) for viral protein. All tissues analyzed in these experiments were collected at Day 34 post-infection, and this information has been added to the Fig 2 legend (Line 221). To further clarify the extent of viral detection, negative-sense viral RNA was detected in lymph nodes from four sheep and the spleen from one sheep, positive-sense viral RNA was detected in lymph nodes from four sheep, and viral protein was detected in a lymph node from one sheep (Lines 222 – 224). A summary of these findings across all tissues examined has been provided in Table C (Page 9) in S1 File.

Reviewer #3: See my comments.

**Conclusions**


-Are the conclusions supported by the data presented?

-Are the limitations of analysis clearly described?

-Do the authors discuss how these data can be helpful to advance our understanding of the topic under study?

-Is public health relevance addressed?

Reviewer #1: Please see "Summary and General Comments".

Reviewer #2: the authors provide an interesting discussion and propose two mechanisms how persistence may occur.
How closely did the sheep experiment mimic a natural infection from tick-bite? route? dose? strain of virus? preparation of inoculum (cell culture, passage history)? age of sheep, how mature is the immune response at this age compared to adult?

**Response:**

We thank the reviewer for this insightful comment. CCHFV persistence was investigated using tissues collected during our previous experimental infection study, in which four domestic sheep (male and approximately two months old) were infected with the CCHFV Kosovo Hoti strain via both subcutaneous and intravenous routes at a dose of 1.2 × 10^6^ TCID50. While using a clinically relevant CCHFV isolate associated with high pathogenicity in humans, the inoculation route might not faithfully recapitulate the course of natural infection through tick bites and sheep at the young age are immunologically immature compared with adult sheep. Future studies should compare the effects of infection route, dose, viral strain and animal age on long-term outcomes. In the revised manuscript we have added a summary of these experimental infection parameters (Lines 120 – 123) and the related discussion (Supplementary results and discussion in S1 file, page 4).

Reviewer #3: See my comment #3.

**Editorial and Data Presentation Modifications?**


Use this section for editorial suggestions as well as relatively minor modifications of existing data that would enhance clarity. If the only modifications needed are minor and/or editorial, you may wish to recommend “Minor Revision” or “Accept”.

Reviewer #1: Nothing in particular.

Reviewer #2: Please see above for suggestions.

Reviewer #3: (No Response)

**Summary and General Comments**


Use this section to provide overall comments, discuss strengths/weaknesses of the study, novelty, significance, general execution and scholarship. You may also include additional comments for the author, including concerns about dual publication, research ethics, or publication ethics. If requesting major revision, please articulate the new experiments that are needed.

Reviewer #1: Missing data and basic errors in the manuscript are evident throughout. Therefore, this manuscript should be appropriately rewritten.

**Response:**

We thank the reviewer for the important feedback. The present study on CCHFV RNA persistence was conducted using archived tissues collected during our previous experimental infection study in sheep [Li et al., 2024; PMID: 38189080]. Although this publication was cited, we acknowledge that the connection between the current study and the previous work was not sufficiently described, which may have contributed to gaps in context and interpretation. In response to the reviewer’s comments, we have revised the manuscript extensively to provide the necessary experimental background, clarify sample origins and study design, and improve the overall presentation and clarity of the manuscript.

Major points
１． No information regarding the viral infection experiment in sheep is provided anywhere. Essential details such as the number of sheep, their age and sex, the inoculation route, and the virus dose are required. If samples from the previous study were used, it is necessary to clearly specify which samples were used.

**Response:**

We thank the reviewer for this important suggestion. As noted above, the present study utilized archived tissues collected during our previous experimental infection study [Li et al., 2024; PMID: 38189080]. To provide appropriate context, a brief description of the essential details for the viral infection experiment in sheep has been added to the revised manuscript (Lines 120 – 123) and is also reproduced below for ease of review:

“CCHFV persistence was investigated using tissues collected during our recent experimental infection study, in which four domestic sheep (male and approximately two months old) were infected with the CCHFV Kosovo Hoti strain via both subcutaneous and intravenous routes at a dose of 1.2 × 10^6^ TCID50 [Ref]”.

In addition, we have included a schematic overview of viremia dynamics observed in these animals (Fig 1A) to provide biological context for the subsequent investigation of post-acute viral RNA persistence.

Samples used in the present study were lymphoid tissues that had exhibited the highest levels of viral RNA in our previous experimental infection study based on RT-qPCR detection of a 57-nt sequence of the S segment [Li et al. 2024. PMID 38189080]. Specifically, four sheep lymph node tissues were used to recover full-length viral genomic RNA sequences by RT-PCR, including Sheep 1 deep cervical lymph nodes (LN), Sheep 2 inguinal LN, Sheep 3 gastrohepatic LN and Sheep 4 inguinal LN (Fig 1 legend, Lines 150 – 152). These tissues were also used in tissue staining experiments for viral RNA and protein, together with additional lymph node and spleen tissues (Fig 2 legend, Lines 222 – 225 and Table C in S1 File, Page 9).

２． From how many samples were the data in Figure 1 obtained? Figure 1A looks like the data from a single sample, since statistical analyses such as standard deviation (SD) are not provided.

**Response:**

Fig 1A is intended as a graphic overview of the previous experimental infection study involving samples from four sheep and serves as background information for the present investigation of viral RNA persistence in tissues. Detailed analyses of animal-to-animal variation and associated statistical parameters were presented in the original publication [Li et al. 2024. PMID 38189080]. For the purposes of the current manuscript, we reanalyzed the data and displayed only the mean viremia values across all four sheep to provide a simplified illustration of the timing and duration of detectable viremia. Since the figure is intended primarily as contextual information rather than as a formal analysis of viremia kinetics, standard deviation values were omitted to improve clarity and readability (Fig 1A).

３． The figure legends are totally too long. It should be described more concisely. For example, the description in Lines 316-320 (Figure 1) is the results and should be described in the Results section of the main text, but not in the figure legend. In addition, there is no data showing this in Figure 1.

**Response:**

We have revised the figure legends by removing interpretive comments and limiting the legends to concise descriptions necessary for understanding the figures (Fig 1, Lines 137 – 155; and Fig 2, Lines 215 – 225).

４． Figures 2, 3 and 4: It is not necessary to present the full-length nucleotide sequence. This is a waste of space. Only the key sites should be shown in a figure or table. Shaded nucleotide indicating nucleotide change should be indicated using the actual nucleotides rather than R, Y, or S in a figure or table.

**Response:**

We have converted these figures into one table showing only the key sites with mutations (Table B in S1 file, Page 8), with degenerate nucleotides indicated using the actual, non-degenerate codes.

５． “Viral RNA persistence in sheep tissues following acute CCHFV infection” has already been reported in the authors’ previous publication. So, the title of this manuscript is not appropriate. The title should reflect the novel findings of this study.

**Response:**

We thank the reviewer for this insightful comment and agree that the original title did not precisely reflect the novel findings of the present study. While our previous publication reported the detection of partial sequences of the S segment, the current work extends those findings by demonstrating the persistence of full-length viral genomic RNA sequences and replication-associated RNA transcripts and proteins. Accordingly, we have revised the title to:

“Persistence of viral full-length genomic RNA sequences and replication-associated RNA transcripts and proteins in sheep tissues following acute Crimean-Congo hemorrhagic fever virus infection”

６． Ultimately, the mechanism underlying the long-term persistence of viral RNA remains unclear. It may be worthwhile to examine the lymph nodes by electron microscopy to determine whether viral particles can be detected.

**Response:**

We thank the reviewer for this valuable comment. In response, we have performed electron microscopy (EM) analyses using the Sheep 4 inguinal lymph node (Supplementary methods and Fig A in S1 File, Pages 5 and 11, respectively). This lymph node tested positive for both viral RNA (negative-sense and positive-sense) and protein in tissue staining experiments (Table C in S1 File, Page 9) and was selected as the most promising target for EM investigation. Two samples from two different areas of the lymph node were processed to generate two blocks of embedded tissue for each area, respectively. One block from each tissue sample was sectioned and stained for observation. 10 grids of each block were sectioned for each block, and four grids of each sample were examined over a period of several days to complete the project. The EM observations did not reveal any detectible virus-like particles.

These data are consistent with the lack of culturable infectious virus (Lines 188 – 190) and the sporadic presence and distribution patterns of viral RNA and protein (Lines 204 – 206). Together, these observations support a model of viral RNA persistence without the extensive and abundant production of infectious virions (Lines 181 – 190). We also note that these results are consistent with the inherent sensitivity limitations of EM. While EM is a valuable tool for morphological confirmation when viral burdens are high, it is generally not well suited for detecting rare or sporadically distributed particles since only a very small fraction of the tissue can be examined at ultrastructural resolution. Nevertheless, we believe these negative EM findings provide useful complementary evidence and therefore have included them in the revised manuscript (Results section, Lines 189 – 190; Supporting information: Supplementary methods and Fig A in S1 File, Pages 5 and 11, respectively). In addition, we would like to include a new coauthor, Andre Dufresne, in recognizing his contribution of the EM investigation.

７． Lines 612-627: Although the possibility of viral reactivation is mentioned, it is unclear whether the authors attempted to investigate this phenomenon. If such attempts were made, the results should be presented and discussed. Otherwise, an extended discussion on this point may not be meaningful.

**Response:**

We thank the reviewer for this important comment. We have not performed experiments specifically designed to investigate viral reactivation. We anticipate that such studies would require viable host cells capable of supporting renewed viral replication. The archived tissues used in the present study have undergone freeze-thaw cycles and are therefore unlikely to be suitable for this purpose. We intend to investigate the potential for CCHFV reactivation in future studies in animals or in vitro co-culture systems with freshly isolated tissues or cells carrying viral RNA persistence. The purpose of the original discussion was not to imply that reactivation had been assessed in the present study, but rather to summarize relevant findings from other viral persistence systems and highlight potential experimental approaches that could be applied to CCHFV in future investigations. To improve the focus and clarity of the manuscript, we have removed this discussion from the main text and relocated it to the Supporting Information (Supplementary results and discussion in S1 File, Page 3 last paragraph). We respectfully submit that this information remains valuable for readers interested in future directions and therefore request its retention in the supplementary file.

Reviewer #2: This is an interesting paper but I found it difficult to navigate. I did access the previous paper describing the methods but this current paper lacks methodology on the sheep experiment. I was unsure if the samples were obtained from the experiment or if it was repeated, it is not clear. I assume it was the same. that does need to be clarified. It is unfortunate the two articles could be published together and providing some brief methodology will provide clarity.

**Response:**

We thank the reviewer for these helpful suggestions, which have significantly improved the clarity and contextualization of the manuscript. We agree that the relationship between the present study and our previous sheep infection study was not sufficiently explained in the original submission. To address this concern, we have provided brief descriptions of the previous sheep infection study, from which archived tissue samples were used in the current paper. These have included the descriptions of the methodology. We agree with the reviewer that it would have been advantageous to publish both studies together. However, we had been unable to conduct in-depth investigation into viral RNA persistence during the original sheep infection study due to technical challenges. The findings reported in the present study were made possible only by the establishment of new methods following tremendous efforts in technical development subsequent to the original animal experiment.

Reviewer #3: The present manuscript by Li H et al. reported successful rescue of full-length virus RNA sequences from tissue samples of sheep persistently infected with a highly pathogenic tick-borne bunyavirus, CCHFV. The significance of the present study is development of a PCR-based sequencing method for demonstrating the existence of complete full-length RNAs of M and S segments of CCHFV even in tissues where only low copy numbers of virus RNAs could be detected. While the reported highly sensitive sequencing method and the results are important for future studies of related pathogens, the present form of the manuscript contains several concerns as follows.

**Response:**

We thank the reviewer for recognizing the significance of this study. We are particularly encouraged by the acknowledgment of the methodological advances reported here, as the work required substantial technical development to investigate a previously unexplored aspect of CCHFV biology. We also sincerely appreciate the reviewer’s insightful and constructive comments, which have helped us improve both the rigor of our interpretations and the clarity of data presentation throughout the manuscript.

Major concern
1) In my understanding, bunyavirus mRNA does not have poly(A) tail because it replicates in the cytoplasm where polyadenylation-related proteins are not present. The authors seem to be convinced that CCHFV mRNA has poly(A) (e.g. Fig. 1A) and performed an experiment to detect poly-A-tailed mRNA (Fig. 6). The authors must need to demonstrate the existence of poly-A-tailed CCHFV mRNA if they would like to adhere to their own model/hypothesis. If the authors withdraw the poly-A-tailed mRNA-based model and experiments, the manuscript must be revisited substantially.

**Response:**

We thank the reviewer for sharing the in-depth molecular biology of bunyavirus mRNA processing and the extremely constructive comment. In response, we have substantially revised our interpretation of the data derived from oligo(dT)-based RNA enrichment. Specifically, we no longer interpret these findings as evidence that CCHFV mRNAs possess bona fide poly(A) tails. The corresponding updated results and discussion have been moved to Supporting information (Supplementary results and discussion in S1 File, Page 2) and are also reproduced below for ease of review. The related methods and figure have also been moved to Supporting information (Supplementary methods in S1 File, Page 5 and Fig G in S1 File, Page 17, respectively). In addition, we have removed the poly(A) notation from all schematic representations of CCHFV mRNA (Fig 1A; and Fig E in S1 File, Page 15).

“Detection of CCHFV sequences in poly(A)-selected RNA pull-downs

We investigated whether CCHFV mRNA was present in sheep tissues harboring persistent viral genomic RNA. Most eukaryotic mRNAs, as well as many viral mRNAs, possess a poly(A) tail [2], which enables affinity purification via oligo(dT) pull-down methods [3, 4]. Although bunyavirus mRNAs are generally considered non-polyadenylated [5], hantavirus M-segment mRNAs have been reported to acquire poly(A) tails using a poly(U) transcription termination signal [6]. Concerning CCHFV, the 3′-terminal processing of its mRNAs has not been well characterized [7].

Using oligo(dT)-based enrichment, we did not detect CCHFV poly(A)-containing RNA in sheep tissues collected at 34 DPI. In contrast, parallel pull-downs from infected cell cultures yielded detectable CCHFV sequences corresponding to all three genomic segments (S, M and L) (Fig G in S1 File). Taken at face value, these findings might suggest that polyadenylated CCHFV RNA is present during active replication in cell culture but absent in persistently infected sheep tissues.

However, a more cautious interpretation is warranted: Re-examination of CCHFV genomic sequences did not reveal canonical polyadenylation signals, including the poly(U) polyadenylation motif described for hantaviruses [2, 6]. Together with the prevailing evidence that bunyavirus mRNAs are typically non-polyadenylated [5], these observations argue against the existence of bona fide poly(A) tails on CCHFV transcripts. Instead, the detection of CCHFV sequences in oligo(dT) pull-downs from the viral RNA-rich cell culture extracts is more plausibly explained by indirect or artifactual mechanisms. These may include co-purification of viral RNA with host poly(A) RNAs within ribonucleoprotein complexes [3, 4], or nonspecific retention and carryover of viral RNA due to incomplete washing or bead-associated background binding. Such effects would be expected to be more pronounced in samples with high viral RNA abundance, such as actively infected cell cultures.”

2) Non-specific priming of reverse transcription reaction has been long discussed in this field (see PMID: 22482442, PMID: 21185869, etc...). The biggest reason of this non-specific priming is the low annealing temperature of RT, and the authors also use the commonly used low temperature, 4 degrees. The amplification observed using culture supernatant (Culture late of Fig. 5B), in which majority of RNA should be negative strand, supports my concern. Thus, the specificity of the RT-nested PCR targeting strand polarity of CCHFV RNA in the present study should be carefully monitored in multiple ways. For example, positive-sense only template in addition to S-IVT should be added to the assay.

**Response:**

We thank the reviewer for drawing our attention to these important studies addressing non-specific priming of reverse transcription (RT). We agree with the reviewer and the reference with PMID 22482442 (Feng et al. 2012) that the RT temperature is an important factor contributing to non-specific priming in strand polarity-targeting RT-PCR. In the original version of this manuscript, non-specific priming of RT occurred in our positive strand-targeting RT-PCR for S, M and L segments when an RT temperature of 50°C was used. In the revised manuscript, we further increased the RT temperature to 65°C. For the S segment, similar results were obtained with non-specific priming remaining detectable even at 65°C (Fig F panel b in S1 File, Page 16), while this temperature resulted in ineffective detection of the M and L sequences in tissues. These results suggest that even higher RT temperatures may be needed to eliminate the observed non-specific priming (however, to our knowledge the 65°C temperature is in a borderline range for RT enzymes), or that other factors in addition to the RT temperature contribute to this issue. We have included updates related to the RT temperatures in the revised manuscript (Supplementary methods in S1 File, Page 5; Table D in S1 File, Page 10; and Fig F panel b legend in S1 File, Page 16).

We have also evaluated the use of nonviral sequence-tagged primers according to the reference with PMID 21185869 (Kawakami et al., 2011). However, this approach failed to generate detectable products from tissue samples and produced non-specific amplicons of incorrect sizes from cell culture samples.

We agree with the reviewer that testing positive-sense only template would provide an additional positive control confirming the capability of the positive strand-targeting RT-PCR for detecting positive-sense RNA. However, such an experiment still would not solve the interference arising from non-specific priming on abundant negative-sense RNA templates which impairs the specificity of positive-sense detection. As a result, the key limitation affecting interpretation of strand-specific RT-PCR would remain unresolved.

Our overall observations from the assessment of strand-specific RT- PCR seem to be consistent with those from another key reference investigating non-specific priming of RT, Tuiskunen et al., 2010 with PMID 19940062. In that study, the authors demonstrated that false priming of RT independent of any primer addition was general to all the various RNAs tested. To overcome the impairment in strand-specific detection of dengue virus RNA, the authors tested several alternative strategies including tagged primers, which were all unsuccessful. As the authors stated, “The search for replicative intermediates of flaviviruses therefore remains unsolved, and all protocols published to date remain to some extent unsatisfying.”. Taken together, both previous and current findings suggest that RT-based strand-specific detection methods may be useful in certain experimental settings but remain vulnerable to substantial false-priming artifacts. For this reason, we consider the strand-targeting RT-PCR data in this study primarily exploratory and place greater emphasis on alternative approaches that do not rely on RT, including in situ hybridization and immunohistochemistry for the detection of replication-associated viral RNA and protein. We therefore respectfully request that the strand-targeting RT-PCR analyses be considered acceptable in their present form.

3) This reviewer strongly recommends the authors to remove the half of the discussion section, in which the present study has not directly contributed. Specifically, the discussions about the mechanism of CCHFV persistent infections (fourth paragraph), reactivation (fifth), association with host health status (sixth), public health importance due to secretion of infectious virus (seventh), and new approach for virus detection (eighth) are not supported by the results newly introduced in the present study. Thus, shortening the discussion section would help readers not to be mislead but to focus on the significance of the present study.

**Response:**

We appreciate this suggestion and agree that the Discussion should remain focused on findings directly supported by the present study. Accordingly, we have removed the discussions addressing mechanisms of persistence, viral reactivation, potential effect on host health status, public health implications and future diagnostic applications from the main manuscript and transferred them to the Supporting Information (Supplementary results and discussion in S1 File, Page 3). Our intention in including these topics was to provide readers with broader context from related viral persistence systems and to outline potential directions for future investigation. We believe these perspectives may still be valuable to interested readers and therefore respectfully request their retention within the supplementary file while keeping the main Discussion focused on the data presented in this study.

Minor concern
4) L. 317-320. These sentences are about the previous results and not associated with the current figure. Please remove.

**Response:**

These interpretive comments have been removed from the Fig 1A legend.

5) L. 327-330. The semi-nested PCR used in the present study to amplify full-length cDNAs of M and S segment RNAs is not actually “nested” since all the primers used bind to the same regions of RNAs/cDNA and there are no “internal primers”. This may need to be called “repeated PCR”, in example, using a mixture of primer pairs with varied length but binding to the same regions.

**Response:**

We thank the reviewer for this insightful comment. We agree with the reviewer that the RT-PCR assay (Fig 1B) differs from a traditional RT-nested PCR since the primer sets partially overlap toward the 5’ ends and do not employ completely internal primers. Nevertheless, the design was based on the concept of nesting, in that the 3′ regions of the second-round primers are positioned internally relative to those used in the first round. A similar principle also applies to the mixtures of primers with staggered 3′ termini used within individual PCR rounds. To better reflect this essential nature of the assay while acknowledging its distinction from classical nested PCR, we have revised the terminology to describe the approach as a “nested-like PCR”. We agree with the reviewer that the assay is also characterized by repeated PCR rounds. Therefore, we have updated the descriptions of the assay as nested-like repeated PCR (Fig 1B legend, Lines 140 – 144; Methods, Lines 263, 270 and 278).

6) L. 367. According to the materials and methods section, the authors made “a 75% majority consensus sequence” based on sequencing reads obtained. But, in the indicated sequences, there are degenerative nucleotides without any criteria to determine. Description to employ the degenerative nucleotides to the positions should be added to the materials and methods section.

**Response:**

We thank the reviewer for this careful observation. A nucleotide position was assigned a non-degenerate base when a single nucleotide accounted for ≥75% of the sequencing reads at that position. If no nucleotide reached this 75% threshold, i.e., two nucleotides both fell within a frequency range of 25% - 75% (exclusive), the position was designated using a degenerate nucleotide code to reflect sequence variation. We have added this clarification to the Methods section (Lines 353 – 357 and 368 – 369).

7) Fig. 2-4 should be summarized to a table and the fasta file should be attached as a supplement.

**Response:**

The sequence data previously presented as Figs 2 – 4 have been condensed into a summary table highlighting only the variable nucleotide positions (Table B in S1 File, Page 8).

8) Fig. 7-9 should be summarized to a single figure because the content is repeat of Fig. 7. Furthermore, the positive cells in Fig. 8 should be indicated by arrowheads. Please clearly indicate that these tissues are from sheep infected with CCHFV at 34 dpi.

**Response:**

We thank the reviewer for this helpful suggestion, which has improved the clarity and conciseness of data presentation. The previous Figs 7 – 9 have been consolidated into a single figure in the revised manuscript (Fig 2, Lines 213 – 225). Positive cells are now indicated by arrowheads in the enlarged panels (Fig 2C, F and I). The figure legend has been revised to clearly indicate that these tissues are from sheep infected with CCHFV at 34 dpi (Line 221).
