# Supplementary material for: Persistence of viral full-length genomic RNA and replication-associated RNA transcripts and proteins in sheep tissues following acute Crimean-Congo hemorrhagic fever virus infection
Source: PLoS Negl Trop Dis. 2026 Oct 30;20(10):e0014842. doi: 10.1371/journal.pntd.0014842 (PMC13734597; doi:10.1371/journal.pntd.0014842)
Supplement: Supplementary file 4 [file pntd.0014842.s004.docx]

**Response to Reviewers**

Reviewer's Responses to Questions

**Key Review Criteria Required for Acceptance?**


As you describe the new analyses required for acceptance, please consider the following:


**Methods**


-Are the objectives of the study clearly articulated with a clear testable hypothesis stated?

-Is the study design appropriate to address the stated objectives?

-Is the population clearly described and appropriate for the hypothesis being tested?

-Is the sample size sufficient to ensure adequate power to address the hypothesis being tested?

-Were correct statistical analysis used to support conclusions?

-Are there concerns about ethical or regulatory requirements being met?

Reviewer #1: The revised manuscript has been appropriately revised in response to my comments. I have no further revisions to request.

Reviewer #2: (No Response)

Reviewer #3: N/A

**Results**


-Does the analysis presented match the analysis plan?

-Are the results clearly and completely presented?

-Are the figures (Tables, Images) of sufficient quality for clarity?

Reviewer #1: The revised manuscript has been appropriately revised in response to my comments. I have no further revisions to request.

Reviewer #2: (No Response)

Reviewer #3: N/A

**Conclusions**


-Are the conclusions supported by the data presented?

-Are the limitations of analysis clearly described?

-Do the authors discuss how these data can be helpful to advance our understanding of the topic under study?

-Is public health relevance addressed?

Reviewer #1: The revised manuscript has been appropriately revised in response to my comments. I have no further revisions to request.

Reviewer #2: (No Response)

Reviewer #3: N/A

**Editorial and Data Presentation Modifications?**

Use this section for editorial suggestions as well as relatively minor modifications of existing data that would enhance clarity. If the only modifications needed are minor and/or editorial, you may wish to recommend “Minor Revision” or “Accept”.

Reviewer #1: Accept

Reviewer #2: (No Response)

Reviewer #3: 1) During the last revision, the authors made substantial removals of results and discussions from the present manuscript but decided to retain many of them as Supporting information (S1File.docx). In my point of view, these changes made the manuscript more difficult to understand because of lacking sufficient rationale to show some results as SI and also to discuss in SI. Minimizing SI only for negative results and large methodological information will be essential to keep the manuscript worth for publication. The results and discussion sections should not rely on SI for fulfilling the authors' claims.

**Response:**

**We thank the reviewer for this important comment. In response, we have further revised the Supporting information (S1 File) and removed the sections that the reviewer previously recommended for deletion during the first round of review, including discussions on the mechanism of CCHFV persistent infections, reactivation, association with host health status, public health importance due to secretion of infectious virus, and new approach for virus detection (S1 File, Pages 2 – 3). We appreciate that these revisions have helped streamline the manuscript and place greater emphasis on the findings and significance of the present study, thereby improving the clarity, focus and overall quality of the manuscript.**

2) Title: "persistence of sequences" sounds incorrect since sequences are not substantial material. "persistence of genomic RNA" should be more precise.

**Response:**

**We have made the suggested modification and appreciate that this has improved the precision of the title.**

3) Fig. 1A: The label of vertical axis contains garbled character (perhaps, micro?).

**Response:**

**We thank the reviewer for identifying this error. The intended character was µ (micro), which was not rendered correctly in the original figure. To avoid potential font-encoding issues associated with Greek characters during figure preparation in Adobe Illustrator, we have converted the reported values to cp/ml. The corrected y-axis label is now shown in the revised Fig. 1A.**

4) Fig. 1A: I assume better to replace the schematic diagram of viral replication in tissues with the actual viral RNA detection results from the previous article.

**Response:**

**We thank the reviewer for this valuable suggestion. Accordingly, we have replaced the schematic diagram of viral replication in tissues with the actual viral RNA detection results from the previous article (updated Fig. 1A, Lines 136 – 139). We agree that this revision provides a more informative representation of the experimental background and improves the presentation of the data.**

5) FIg. 1D: "Genomic RNA" should be removed because these bands are not genomic RNA itself.

**Response:**

**We thank the reviewer for this comment. We agree that these bands are not genomic RNA itself and have therefore removed the "Genomic RNA" label from Fig. 1D accordingly.**

**Summary and General Comments**

Use this section to provide overall comments, discuss strengths/weaknesses of the study, novelty, significance, general execution and scholarship. You may also include additional comments for the author, including concerns about dual publication, research ethics, or publication ethics. If requesting major revision, please articulate the new experiments that are needed.

Reviewer #1: The revised manuscript has been appropriately revised in response to my comments. I have no further revisions to request.

Reviewer #2: the authors have made appropriate revisions for the comments I submitted.

Reviewer #3: I respect the authors to respond my comments in honest manner and their attitude to try to follow reviewers' suggestions as much as possible but also want to retain their claims. However, moving many parts of the previous manuscript on which this reviewer criticized in the last review, away from the main body but keeping them in SI may not be the better way for constructing a scientific paper. I hope the authors can make their substantial efforts on revising this manuscript in more appropriate style.

**Response:**

**We thank the reviewer for the encouraging comments in recognizing our efforts to address the reviewers’ suggestions in a transparent and respectful manner. Our request to retain those supplementary sections in SI was motivated by a desire to provide a broader context from related viral persistence systems that may be of value to interested readers. However, upon further consideration, we have now removed these sections (S1 File, Pages 2–3), as we agree that these revisions improve the overall organization, clarity and scientific focus of the manuscript. We are grateful for the reviewer’s rigorous evaluation and constructive recommendations, which have substantially strengthened the manuscript.**
