# Supplementary material for: Persistence of viral full-length genomic RNA and replication-associated RNA transcripts and proteins in sheep tissues following acute Crimean-Congo hemorrhagic fever virus infection
Source: PLoS Negl Trop Dis. 2026 Oct 30;20(10):e0014842. doi: 10.1371/journal.pntd.0014842 (PMC13734597; doi:10.1371/journal.pntd.0014842)
Supplement: Peer review history

# Peer review: Decision Letter 0

**DOI:** 10.1371/journal.pntd.0014842.r001

**Authors:** Ayato Takada, Stephanie N. Seifert

29 Apr 2026

Viral RNA persistence in sheep tissues following acute Crimean-Congo hemorrhagic fever virus infection

PLOS Neglected Tropical Diseases

Dear Dr. Pickering,

Thank you for submitting your manuscript to PLOS Neglected Tropical Diseases. After careful consideration, we feel that it has merit but does not fully meet PLOS Neglected Tropical Diseases's publication criteria as it currently stands. Therefore, we invite you to submit a revised version of the manuscript that addresses the points raised during the review process.

Please submit your revised manuscript within by Jun 28 2026 11:59PM. If you will need more time than this to complete your revisions, please reply to this message or contact the journal office at plosntds@plos.org. When you're ready to submit your revision, log on to https://www.editorialmanager.com/pntd/ and select the 'Submissions Needing Revision' folder to locate your manuscript file.

Please include the following items when submitting your revised manuscript:

* A letter that responds to each point raised by the editor and reviewer(s). You should upload this letter as a separate file labeled 'Response to Reviewers'. This file does not need to include responses to any formatting updates and technical items listed in the 'Journal Requirements' section below.

* A marked-up copy of your manuscript that highlights changes made to the original version. You should upload this as a separate file labeled 'Revised Manuscript with Track Changes'.

* An unmarked version of your revised paper without tracked changes. You should upload this as a separate file labeled 'Manuscript'.

If you would like to make changes to your financial disclosure, competing interests statement, or data availability statement, please make these updates within the submission form at the time of resubmission. Guidelines for resubmitting your figure files are available below the reviewer comments at the end of this letter.

We look forward to receiving your revised manuscript.

Kind regards,

Ayato Takada, Ph.D.

Academic Editor

PLOS Neglected Tropical Diseases

Stephanie Seifert

Section Editor

PLOS Neglected Tropical Diseases

Shaden Kamhawi

co-Editor-in-Chief

PLOS Neglected Tropical Diseases

orcid.org/0000-0003-4304-636XX

Paul Brindley

co-Editor-in-Chief

PLOS Neglected Tropical Diseases

orcid.org/0000-0003-1765-0002

**Journal Requirements:**

If the reviewer comments include a recommendation to cite specific previously published works, please review and evaluate these publications to determine whether they are relevant and should be cited. There is no requirement to cite these works unless the editor has indicated otherwise.

1) Please ensure that the CRediT author contributions listed for every co-author are completed accurately and in full.

At this stage, the following Authors/Authors require contributions: Hongzhao Li, Estella Moffat, Katherine Handel, Michelle Nebroski, Daniel Sullivan, Carissa Embury-Hyatt, Oliver Lung, and Bradley S Pickering. Please ensure that the full contributions of each author are acknowledged in the "Add/Edit/Remove Authors" section of our submission form.

The list of CRediT author contributions may be found here: https://journals.plos.org/plosntds/s/authorship#loc-author-contributions

2) We ask that a manuscript source file is provided at Revision. Please upload your manuscript file as a .doc, .docx, .rtf or .tex. If you are providing a .tex file, please upload it under the item type u2018LaTeX Source Fileu2019 and leave your .pdf version as the item type u2018Manuscriptu2019.

3) We noticed that you used the phrase 'data not shown' in the manuscript. We do not allow these references, as the PLOS data access policy requires that all data be either published with the manuscript or made available in a publicly accessible database. Please amend the supplementary material to include the referenced data or remove the references.

4) We do not publish any copyright or trademark symbols that usually accompany proprietary names, eg ©,  ®, or TM  (e.g. next to drug or reagent names). Therefore please remove all instances of trademark/copyright symbols throughout the text, including:

- ® on page: 19.

5) Your manuscript's sections are not in the correct order.  Please amend to the following order: Abstract, Introduction, Results, Discussion, and Methods

6) Please upload all main figures as separate Figure files in .tif or .eps format. For more information about how to convert and format your figure files please see our guidelines:

https://journals.plos.org/plosntds/s/figures

7) Please amend your detailed Financial Disclosure statement. This is published with the article. It must therefore be completed in full sentences and contain the exact wording you wish to be published.

1) State the initials, alongside each funding source, of each author to receive each grant. For example: "This work was supported by the National Institutes of Health (####### to AM; ###### to CJ) and the National Science Foundation (###### to AM)."

2) State what role the funders took in the study. If the funders had no role in your study, please state: "The funders had no role in study design, data collection and analysis, decision to publish, or preparation of the manuscript."

3) If any authors received a salary from any of your funders, please state which authors and which funders..

If you did not receive any funding for this study, please simply state: u201cThe authors received no specific funding for this work.u201d

**Reviewers' Comments:**

Reviewer's Responses to Questions

**Key Review Criteria Required for Acceptance?**

As you describe the new analyses required for acceptance, please consider the following:

**Methods**

-Are the objectives of the study clearly articulated with a clear testable hypothesis stated?

-Is the study design appropriate to address the stated objectives?

-Is the population clearly described and appropriate for the hypothesis being tested?

-Is the sample size sufficient to ensure adequate power to address the hypothesis being tested?

-Were correct statistical analysis used to support conclusions?

-Are there concerns about ethical or regulatory requirements being met?

Reviewer #1: Please see "Summary and General Comments".

Reviewer #2: The authors could make it clearer that this paper investigated the characteristics of CCHFV RNA in lymphoid tissues from which complete genome sequence was previously obtained. This is mentioned in the results but would be useful to include in the objectives. Similarly that there were technical difficulties previously that the researchers used several methods overcome.

The authors refer to a related paper for the experimental infection of the sheep. It was also not clear if the samples were obtained from the previous experiment although I assumed they were due to the similarity of sampling intervals etc. I understand the authors do not want to repeat the entire methodology however I strongly suggest that a summary of the sheep experiments be included. It was not possible to evaluate this paper without accessing the previous publication. The authors presented evidence that viral whole genome RNA sequences persist in tissues after acute viremia, and detected replication associated viral RNA transcripts and viral proteins. The authors could briefly provide the experimental approach to assist with understanding the results presented in this paper. Where did the primers come from? There is an extensive table, were these all designed in house and what strategy?

The methods describe the various RT-nested PCR reactions to amplify viral RNA, genomic RNA and targeting strand polarity of CCHFV RNA, perhaps these could be summarised and presently more succinctly using tables.

Reviewer #3: See my comments #1 and #2.

**********

**Results**

-Does the analysis presented match the analysis plan?

-Are the results clearly and completely presented?

-Are the figures (Tables, Images) of sufficient quality for clarity?

Reviewer #1: Please see "Summary and General Comments".

Reviewer #2: It is unclear exactly what tissues were positive and what tissues were homogenised for RNA extraction. A brief summary is needed. Moving some of the comments in results to methodology may assist.

The authors may want to consider removing "comments" from the figure legends and keep to a precise description of the figure.

The sequence data may be more suitable in supplementary data with a summary of the mutations

Fig 7. Were the tissues from day 34?

From how many sheep and tissues was CCHF sequence detected?

Reviewer #3: See my comments.

**********

**Conclusions**

-Are the conclusions supported by the data presented?

-Are the limitations of analysis clearly described?

-Do the authors discuss how these data can be helpful to advance our understanding of the topic under study?

-Is public health relevance addressed?

Reviewer #1: Please see "Summary and General Comments".

Reviewer #2: the authors provide an interesting discussion and propose two mechanisms how persistence may occur.

How closely did the sheep experiment mimic a natural infection from tick-bite? route? dose? strain of virus? preparation of inoculum (cell culture, passage history)? age of sheep, how mature is the immune response at this age compared to adult?

Reviewer #3: See my comment #3.

**********

**Editorial and Data Presentation Modifications?**

Use this section for editorial suggestions as well as relatively minor modifications of existing data that would enhance clarity. If the only modifications needed are minor and/or editorial, you may wish to recommend “Minor Revision” or “Accept”.

Reviewer #1: Nothing in particular.

Reviewer #2: Please see above for suggestions.

Reviewer #3: (No Response)

**********

**Summary and General Comments**

Use this section to provide overall comments, discuss strengths/weaknesses of the study, novelty, significance, general execution and scholarship. You may also include additional comments for the author, including concerns about dual publication, research ethics, or publication ethics. If requesting major revision, please articulate the new experiments that are needed.

Reviewer #1: Missing data and basic errors in the manuscript are evident throughout. Therefore, this manuscript should be appropriately rewritten.

Major points

１． No information regarding the viral infection experiment in sheep is provided anywhere. Essential details such as the number of sheep, their age and sex, the inoculation route, and the virus dose are required. If samples from the previous study were used, it is necessary to clearly specify which samples were used.

２． From how many samples were the data in Figure 1 obtained? Figure 1A looks like the data from a single sample, since statistical analyses such as standard deviation (SD) are not provided.

３． The figure legends are totally too long. It should be described more concisely. For example, the description in Lines 316-320 (Figure 1) is the results and should be described in the Results section of the main text, but not in the figure legend. In addition, there is no data showing this in Figure 1.

４． Figures 2, 3 and 4: It is not necessary to present the full-length nucleotide sequence. This is a waste of space. Only the key sites should be shown in a figure or table. Shaded nucleotide indicating nucleotide change should be indicated using the actual nucleotides rather than R, Y, or S in a figure or table.

５． “Viral RNA persistence in sheep tissues following acute CCHFV infection” has already been reported in the authors’ previous publication. So, the title of this manuscript is not appropriate. The title should reflect the novel findings of this study.

６． Ultimately, the mechanism underlying the long-term persistence of viral RNA remains unclear. It may be worthwhile to examine the lymph nodes by electron microscopy to determine whether viral particles can be detected.

７． Lines 612-627: Although the possibility of viral reactivation is mentioned, it is unclear whether the authors attempted to investigate this phenomenon. If such attempts were made, the results should be presented and discussed. Otherwise, an extended discussion on this point may not be meaningful.

Reviewer #2: This is an interesting paper but I found it difficult to navigate. I did access the previous paper describing the methods but this current paper lacks methodology on the sheep experiment. I was unsure if the samples were obtained from the experiment or if it was repeated, it is not clear. I assume it was the same. that does need to be clarified. It is unfortunate the two articles could be published together and providing some brief methodology will provide clarity.

Reviewer #3: The present manuscript by Li H et al. reported successful rescue of full-length virus RNA sequences from tissue samples of sheep persistently infected with a highly pathogenic tick-borne bunyavirus, CCHFV. The significance of the present study is development of a PCR-based sequencing method for demonstrating the existence of complete full-length RNAs of M and S segments of CCHFV even in tissues where only low copy numbers of virus RNAs could be detected. While the reported highly sensitive sequencing method and the results are important for future studies of related pathogens, the present form of the manuscript contains several concerns as follows.

Major concern

1) In my understanding, bunyavirus mRNA does not have poly(A) tail because it replicates in the cytoplasm where polyadenylation-related proteins are not present. The authors seem to be convinced that CCHFV mRNA has poly(A) (e.g. Fig. 1A) and performed an experiment to detect poly-A-tailed mRNA (Fig. 6). The authors must need to demonstrate the existence of poly-A-tailed CCHFV mRNA if they would like to adhere to their own model/hypothesis. If the authors withdraw the poly-A-tailed mRNA-based model and experiments, the manuscript must be revisited substantially.

2) Non-specific priming of reverse transcription reaction has been long discussed in this field (see PMID: 22482442, PMID: 21185869, etc...). The biggest reason of this non-specific priming is the low annealing temperature of RT, and the authors also use the commonly used low temperature, 4 degrees. The amplification observed using culture supernatant (Culture late of Fig. 5B), in which majority of RNA should be negative strand, supports my concern. Thus, the specificity of the RT-nested PCR targeting strand polarity of CCHFV RNA in the present study should be carefully monitored in multiple ways. For example, positive-sense only template in addition to S-IVT should be added to the assay.

3) This reviewer strongly recommends the authors to remove the half of the discussion section, in which the present study has not directly contributed. Specifically, the discussions about the mechanism of CCHFV persistent infections (fourth paragraph), reactivation (fifth), association with host health status (sixth), public health importance due to secretion of infectious virus (seventh), and new approach for virus detection (eighth) are not supported by the results newly introduced in the present study. Thus, shortening the discussion section would help readers not to be mislead but to focus on the significance of the present study.

Minor concern

4) L. 317-320. These sentences are about the previous results and not associated with the current figure. Please remove.

5) L. 327-330. The semi-nested PCR used in the present study to amplify full-length cDNAs of M and S segment RNAs is not actually “nested” since all the primers used bind to the same regions of RNAs/cDNA and there are no “internal primers”. This may need to be called “repeated PCR”, in example, using a mixture of primer pairs with varied length but binding to the same regions.

6) L. 367. According to the materials and methods section, the authors made “a 75% majority consensus sequence” based on sequencing reads obtained. But, in the indicated sequences, there are degenerative nucleotides without any criteria to determine. Description to employ the degenerative nucleotides to the positions should be added to the materials and methods section.

7) Fig. 2-4 should be summarized to a table and the fasta file should be attached as a supplement.

8) Fig. 7-9 should be summarized to a single figure because the content is repeat of Fig. 7. Furthermore, the positive cells in Fig. 8 should be indicated by arrowheads. Please clearly indicate that these tissues are from sheep infected with CCHFV at 34 dpi.

**********

PLOS authors have the option to publish the peer review history of their article (what does this mean?). If published, this will include your full peer review and any attached files.

If you choose “no”, your identity will remain anonymous but your review may still be made public.

**Do you want your identity to be public for this peer review?**  For information about this choice, including consent withdrawal, please see our Privacy Policy.

Reviewer #1: No

Reviewer #2: No

Reviewer #3: No

**Figure resubmission:**

**Reproducibility:**

To enhance the reproducibility of your results, we recommend that authors of applicable studies deposit laboratory protocols in protocols.io, where a protocol can be assigned its own identifier (DOI) such that it can be cited independently in the future. Additionally, PLOS ONE offers an option to publish peer-reviewed clinical study protocols. Read more information on sharing protocols at https://plos.org/protocols?utm_medium=editorial-email&utm_source=authorletters&utm_campaign=protocols

# Author comment: Author response to Decision Letter 1

**DOI:** 10.1371/journal.pntd.0014842.r002

27 Jul 2026

Attachment Submitted filename: 2026-07-23 Sheep2 Response to Reviewers PNTD.docx

# Peer review: Decision Letter 1

**DOI:** 10.1371/journal.pntd.0014842.r003

**Authors:** Ayato Takada, Stephanie N. Seifert

5 Oct 2026

Persistence of viral full-length genomic RNA sequences and replication-associated RNA transcripts and proteins in sheep tissues following acute Crimean-Congo hemorrhagic fever virus infection

Thank you for submitting your manuscript to PLOS Neglected Tropical Diseases. After careful consideration, we feel that it has merit but does not fully meet PLOS Neglected Tropical Diseases's publication criteria as it currently stands. Therefore, we invite you to submit a revised version of the manuscript that addresses the points raised during the review process.

Please submit your revised manuscript by Dec 04 2026 11:59PM. If you will need more time than this to complete your revisions, please reply to this message or contact the journal office at plosntds@plos.org. When you're ready to submit your revision, log on to https://www.editorialmanager.com/pntd/ and select the 'Submissions Needing Revision' folder to locate your manuscript file.

Response to Reviewers Revised Manuscript with Track Changes

* An unmarked version of your revised paper without tracked changes. You should upload this as a separate file labeled 'Manuscript'.

As the corresponding author, your ORCID iD is verified in the submission system and will appear in the published article. PLOS supports the use of ORCID, and we encourage all coauthors to register for an ORCID iD and use it as well. Please encourage your coauthors to verify their ORCID iD within the submission system before final acceptance, as unverified ORCID iDs will not appear in the published article. Only the individual author can complete the verification step; PLOS staff cannot verify ORCID iDs on behalf of authors.

PLOS Neglected Tropical Diseases

Stephanie Seifert, Ph.D.

Section Editor

PLOS Neglected Tropical Diseases

Shaden Kamhawi

co-Editor-in-Chief

PLOS Neglected Tropical Diseases

orcid.org/0000-0003-4304-636XX

Paul Brindley

co-Editor-in-Chief

PLOS Neglected Tropical Diseases

orcid.org/0000-0003-1765-0002

**Reviewers' comments:**

**Key Review Criteria Required for Acceptance?**

As you describe the new analyses required for acceptance, please consider the following:

**Methods**

-Are the objectives of the study clearly articulated with a clear testable hypothesis stated?

-Is the study design appropriate to address the stated objectives?

-Is the population clearly described and appropriate for the hypothesis being tested?

-Is the sample size sufficient to ensure adequate power to address the hypothesis being tested?

-Were correct statistical analysis used to support conclusions?

-Are there concerns about ethical or regulatory requirements being met?

Reviewer #1: The revised manuscript has been appropriately revised in response to my comments. I have no further revisions to request.

Reviewer #2: (No Response)

Reviewer #3: N/A

**********

**Results**

-Does the analysis presented match the analysis plan?

-Are the results clearly and completely presented?

-Are the figures (Tables, Images) of sufficient quality for clarity?

Reviewer #1: The revised manuscript has been appropriately revised in response to my comments. I have no further revisions to request.

Reviewer #2: (No Response)

Reviewer #3: N/A

**********

**Conclusions**

-Are the conclusions supported by the data presented?

-Are the limitations of analysis clearly described?

-Do the authors discuss how these data can be helpful to advance our understanding of the topic under study?

-Is public health relevance addressed?

Reviewer #1: The revised manuscript has been appropriately revised in response to my comments. I have no further revisions to request.

Reviewer #2: (No Response)

Reviewer #3: N/A

**********

**Editorial and Data Presentation Modifications?**

Use this section for editorial suggestions as well as relatively minor modifications of existing data that would enhance clarity. If the only modifications needed are minor and/or editorial, you may wish to recommend “Minor Revision” or “Accept”.

Reviewer #1: Accept

Reviewer #2: (No Response)

Reviewer #3: 1) During the last revision, the authors made substantial removals of results and discussions from the present manuscript but decided to retain many of them as Supporting information (S1File.docx). In my point of view, these changes made the manuscript more difficult to understand because of lacking sufficient rationale to show some results as SI and also to discuss in SI. Minimizing SI only for negative results and large methodological information will be essential to keep the manuscript worth for publication. The results and discussion sections should not rely on SI for fulfilling the authors' claims.

2) Title: "persistence of sequences" sounds incorrect since sequences are not substantial material. "persistence of genomic RNA" should be more precise.

3) Fig. 1A: The label of vertical axis contains garbled character (perhaps, micro?).

4) Fig. 1A: I assume better to replace the schematic diagram of viral replication in tissues with the actual viral RNA detection results from the previous article.

5) FIg. 1D: "Genomic RNA" should be removed because these bands are not genomic RNA itself.

**********

**Summary and General Comments**

Use this section to provide overall comments, discuss strengths/weaknesses of the study, novelty, significance, general execution and scholarship. You may also include additional comments for the author, including concerns about dual publication, research ethics, or publication ethics. If requesting major revision, please articulate the new experiments that are needed.

Reviewer #1: The revised manuscript has been appropriately revised in response to my comments. I have no further revisions to request.

Reviewer #2: the authors have made appropriate revisions for the comments I submitted.

Reviewer #3: I respect the authors to respond my comments in honest manner and their attitude to try to follow reviewers' suggestions as much as possible but also want to retain their claims. However, moving many parts of the previous manuscript on which this reviewer criticized in the last review, away from the main body but keeping them in SI may not be the better way for constructing a scientific paper. I hope the authors can make their substantial efforts on revising this manuscript in more appropriate style.

**********

PLOS authors have the option to publish the peer review history of their article (what does this mean?). If published, this will include your full peer review and any attached files.

If you choose “no”, your identity will remain anonymous but your review may still be made public.

**Do you want your identity to be public for this peer review?**  For information about this choice, including consent withdrawal, please see our Privacy Policy.

Reviewer #1: No

Reviewer #2: No

Reviewer #3: No

**Figure resubmission:**

**Reproducibility:** -->To enhance the reproducibility of your results, we recommend that authors of applicable studies deposit laboratory protocols in protocols.io, where a protocol can be assigned its own identifier (DOI) such that it can be cited independently in the future. Additionally, PLOS ONE offers an option to publish peer-reviewed clinical study protocols. Read more information on sharing protocols at https://plos.org/protocols?utm_medium=editorial-email&utm_source=authorletters&utm_campaign=protocols-->?>

# Author comment: Author response to Decision Letter 2

**DOI:** 10.1371/journal.pntd.0014842.r004

15 Oct 2026

Attachment Submitted filename: 2026-10-06 Sheep2 Response to Reviewers PNTD.docx

# Editor report: Decision Letter 2

**DOI:** 10.1371/journal.pntd.0014842.r005

**Authors:** Ayato Takada, Stephanie N. Seifert

20 Oct 2026

Dear Dr. Pickering,

We are pleased to inform you that your manuscript 'Persistence of viral full-length genomic RNA and replication-associated RNA transcripts and proteins in sheep tissues following acute Crimean-Congo hemorrhagic fever virus infection' has been provisionally accepted for publication in PLOS Neglected Tropical Diseases.

Before your manuscript can be formally accepted you will need to complete some formatting changes, which you will receive in a follow up email. A member of our team will be in touch with a set of requests.

Please note that your manuscript will not be scheduled for publication until you have made the required changes, so a swift response is appreciated.

IMPORTANT: The editorial review process is now complete. PLOS will only permit corrections to spelling, formatting or significant scientific errors from this point onwards. Requests for major changes, or any which affect the scientific understanding of your work, will cause delays to the publication date of your manuscript.

Should you, your institution's press office or the journal office choose to press release your paper, you will automatically be opted out of early publication. We ask that you notify us now if you or your institution is planning to press release the article. All press must be co-ordinated with PLOS.

Thank you again for supporting Open Access publishing; we are looking forward to publishing your work in PLOS Neglected Tropical Diseases.

Best regards,

Ayato Takada, Ph.D.

Academic Editor

PLOS Neglected Tropical Diseases

Stephanie Seifert

Section Editor

PLOS Neglected Tropical Diseases

Shaden Kamhawi

co-Editor-in-Chief

PLOS Neglected Tropical Diseases

orcid.org/0000-0003-4304-636XX

Paul Brindley

co-Editor-in-Chief

PLOS Neglected Tropical Diseases

orcid.org/0000-0003-1765-0002

***********************************************************

p.p1 {margin: 0.0px 0.0px 0.0px 0.0px; line-height: 16.0px; font: 14.0px Arial; color: #323333; -webkit-text-stroke: #323333}span.s1 {font-kerning: none

# Editor report: Acceptance letter

**DOI:** 10.1371/journal.pntd.0014842.r006

**Authors:** Ayato Takada, Stephanie N. Seifert

Dear Dr. Pickering,

We are delighted to inform you that your manuscript, "Persistence of viral full-length genomic RNA and replication-associated RNA transcripts and proteins in sheep tissues following acute Crimean-Congo hemorrhagic fever virus infection," has been formally accepted for publication in PLOS Neglected Tropical Diseases.

We have now passed your article onto the PLOS Production Department who will complete the rest of the publication process. All authors will receive a confirmation email upon publication.

The corresponding author will soon be receiving a typeset proof for review, to ensure errors have not been introduced during production. Please review the PDF proof of your manuscript carefully, as this is the last chance to correct any scientific or type-setting errors. Please note that major changes, or those which affect the scientific understanding of the work, will likely cause delays to the publication date of your manuscript. Note: Proofs for Front Matter articles (Editorial, Viewpoint, Symposium, Review, etc...) are generated on a different schedule and may not be made available as quickly.

Soon after your final files are uploaded, the early version of your manuscript will be published online unless you opted out of this process. The date of the early version will be your article's publication date. The final article will be published to the same URL, and all versions of the paper will be accessible to readers.

For Research Articles, you will receive an invoice from PLOS for your publication fee after your manuscript has reached the completed accept phase. If you receive an email requesting payment before acceptance or for any other service, this may be a phishing scheme. Learn how to identify phishing emails and protect your accounts at https://explore.plos.org/phishing.

Thank you again for supporting open-access publishing; we are looking forward to publishing your work in PLOS Neglected Tropical Diseases.

Best regards,

Shaden Kamhawi

co-Editor-in-Chief

PLOS Neglected Tropical Diseases

Paul Brindley

co-Editor-in-Chief

PLOS Neglected Tropical Diseases
